# Substrate secondary structure is associated with proteasomal cleavage propensity

**DOI:** 10.3389/fbinf.2026.1730535

**Published:** 2026-10-07

**Authors:** Matheus De Bastos Balbé Gutierres, Finn Beruldsen, Dinler Amaral Antunes, Gustavo Fioravanti Vieira

**Affiliations:** 1 Programa de Pós-Graduação em Genética e Biologia Molecular (PPGBM-UFRGS), Porto Alegre, Brazil; 2 Department of Biology and Biochemistry, University of Houston, Houston, TX, United States; 3 Programa de Pós-Graduação em Saúde e Desenvolvimento Humano – PPGSAUDEH, Universidade La Salle, Canoas, Brazil

**Keywords:** immunoinformatics, immunoproteasome, machine learning (ML), MHC-I, proteasome, secondary structures, T-cell epitopes

## Abstract

Why does the proteasome cleave proteins at some peptide bonds but not others? Prior work has emphasized sequence motifs, amino acid properties, and proteasome isoform, but the role of local secondary-structure context remains unclear. Here, we evaluate whether helix, strand, and coil context help explain proteasomal cleavage and whether such information can improve cleavage-site prediction. By mining a human and viral PDB structure corpus, we found that high-confidence predicted cleavage sites are enriched in beta-strand extended conformations and depleted in coil-like regions. We then trained a machine learning model using only secondary-structure labels, and benchmarked its performance against a sequence-aware baseline, other well-known predictors, and ensemble approaches using hybrid position-specific scoring matrices (PSSMs). Our proof-of-concept SSO-8 model was trained on secondary structure features predicted by NetSurfP-3.0 for 8-mer sequences derived from a diverse experimental digestion dataset of 20 substrate proteins. SSO-8 reached AUC = 0.649 on a held-out benchmark constitutive dataset and AUC = 0.737 on a held-out immunoproteasome dataset. A same-protocol sequence-only model reached AUC of 0.674 and 0.736, respectively, and paired peptide/protein-level tests found no evidence of a significant SSO-8-vs-sequence difference. Our model was also competitive with other sequence-based proteasomal prediction methods, using different training sets and architectures. Therefore, secondary-structure-only information captured substantial cleavage signal despite using much less input information than full substrate sequence, with the clearest support in the immunoproteasome dataset and hybrid PSSM analyses. These results extend the known preference for extended active-site geometry by showing an association between local substrate backbone conformation and proteasomal cleavage propensities, particularly in the context of the immunoproteasome, and suggest the use of sequence-encoded secondary-structure propensities as a measurable feature of proteasomal cleavage preferences.

## Introduction

Proteasomal cleavage is the first step in the endogenous peptide presenting pathway, which is present in most cells, and enables cellular immunity to distinguish cells harboring pathological (“non-self”) from physiological (“self”) proteins (Tenzer et al., 2005). Even small shifts in where peptides are cut from larger proteins can have important implications on the resulting immune responses (Eggers et al., 1995; Toes et al., 2001). A single misplaced cleavage can erase or alter an epitope (*i.e.*, peptide recognized by T cell lymphocytes), potentially tipping the balance between immune escape and immune attack (Beekman et al., 2000). Despite decades of work across disciplines, we still cannot reliably predict where the proteasome will slice any given protein. Uncovering these rules can help refine proteasomal-cleavage and antigen-processing prediction models, with potential applications to epitope selection and immunotherapy design.

The constitutive 26S proteasome is a 2.5 MDa complex made of a 20S catalytic core and 19S regulatory particles: the latter unfold and translocate ubiquitinated substrates in an ATP-dependent manner (Finley, 2009; Dahlmann et al., 1989; Bard et al., 2018; Löwe et al., 1995) (Figure 1). In healthy cells, the constitutive proteasome is involved in protein degradation to maintain cellular protein homeostasis, including the degradation of misfolded proteins, regulation of cellular processes, and generation of peptides for the endogenous peptide presenting pathway (Rousseau and Bertolotti, 2018; Franić et al., 2021; Çetin et al., 2021). Under inflammatory conditions, interferon-γ (IFNγ) induces replacement of the constitutive β1/β2/β5 subunits with immunoproteasome isoforms, which more efficiently generate ligands for the class I major histocompatibility complex (MHCI) receptors (Aki et al., 1994). A narrow (∼15 Å) channel in the α ring restricts entry into the 20S core, forcing substrates to unfold and thread in an extended conformation (Löwe et al., 1995; Förster and Hill, 2003). Classic protease substrate co-crystal structures (Tynd et al., 2005; Madala et al., 2010) suggest that substrates must “flatten” into an extended beta strand conformation within the proteasome’s catalytic groove (Harshbarger et al., 2015). This idea is further supported by the observation that inhibitors such as carfilzomib and bortezomib also mimic an extended beta strand conformation.

**FIGURE 1:**
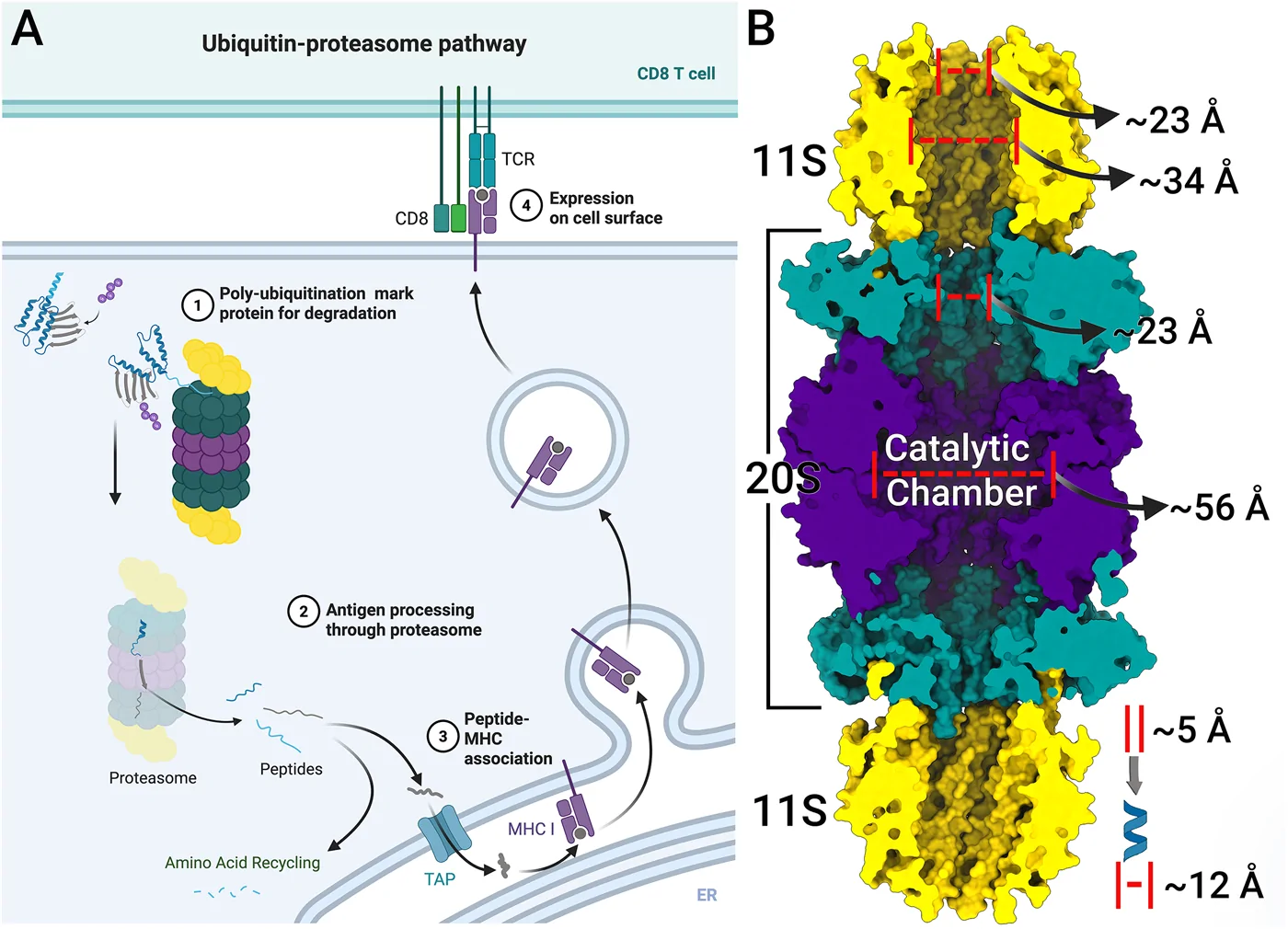
The ubiquitin-proteasome pathway. **(A)** Intracellular proteins are marked for degradation, processed within the proteasome, and the resulting peptides are presented by Class I Major Histocompatibility Complex (MHCI) molecules. The proteasome yellow caps in this schematic represent 19S regulatory particles. Created with BioRender.com. **(B)** Cutaway view of the yeast 20S proteasome bound to PA26, an 11S regulator from Trypanosoma brucei. Yellow caps are PA26/11S, cyan rings are α rings, and purple rings are β rings. The axial channel and proteolytic chamber are visible in the cutaway. Approximate channel dimensions are depicted, in comparison to mean widths of α-helix and extended conformations. The outer opening of the 11S particle narrows to ∼13–23 Å, leading into a chamber with an internal diameter of ∼30–35 Å. The chamber of the 20S core particle measures ∼50–60 Å in diameter. These values represent approximate ranges observed across conformational states of the proteasome complex. Structure obtained from the Protein Data Bank (PDB ID 1FNT), (Protein Data Bank, 2025) and image produced with UCSF ChimeraX (Meng et al., 2023).

It is known that sequence motifs contribute to proteasomal cleavage specificity. For instance, the β5 domain of the proteasome favors hydrophobic residues, while β2 favors basic residues, and immunoproteasome subunits further modulate these preferences (Toes et al., 2001). However, sequence motifs alone do not explain why cleavage is biased toward particular local structural contexts. The key question is how much cleavage signal remains after amino-acid identity is removed.

This work asks whether local substrate backbone conformation leaves a detectable signature for proteasomal cleavage. We tested whether predicted cleavage sites show reproducible secondary-structure preferences, whether structure-only models retain predictive signal, and whether structure-derived evidence adds information to sequence-aware predictors. Across these analyses, secondary-structure information was consistently informative despite its reduced input representation, with strongest support in the immunoproteasome benchmarks.

Our findings identify local backbone state as an interpretable correlate of proteasomal cleavage specificity. This beta-strand-associated signal, stronger for the immunoproteasome, may help explain why some predicted cleavage sites are favored in epitope-discovery models. It may also raise a testable hypothesis connecting immunoproteasome processing with structurally constrained regions, where mutations may be less tolerated. Our study does not provide a mechanistic basis for the observed cleavage biases, but it is possible that productive cleavage requires an engaged substrate segment to access an extended, active-site-compatible conformation. In this context, strand annotations may reflect regions that more readily enter productive protease geometry (Harshbarger et al., 2015; da Fonseca and Morris, 2015). Whether this bias toward structurally constrained regions contributes to epitope conservation or T-cell recognition remains to be tested (Gaiha et al., 2019; Gorin et al., 2017).

## Materials and methods

### Reference datasets

To create a reference protein dataset with labeled secondary structures, we obtained experimentally determined structures of proteins from the Protein Data Bank (Berman et al., 2000). We selected only non-redundant human or viral protein structures at ≤ 2.5 A resolution. After deduplication and additional filtering, the structure corpus contained 1,505 PDB IDs (Figure 2). These proteins are referred to as the PDB corpus (see Supplementary Material for PDB IDs).

**FIGURE 2:**
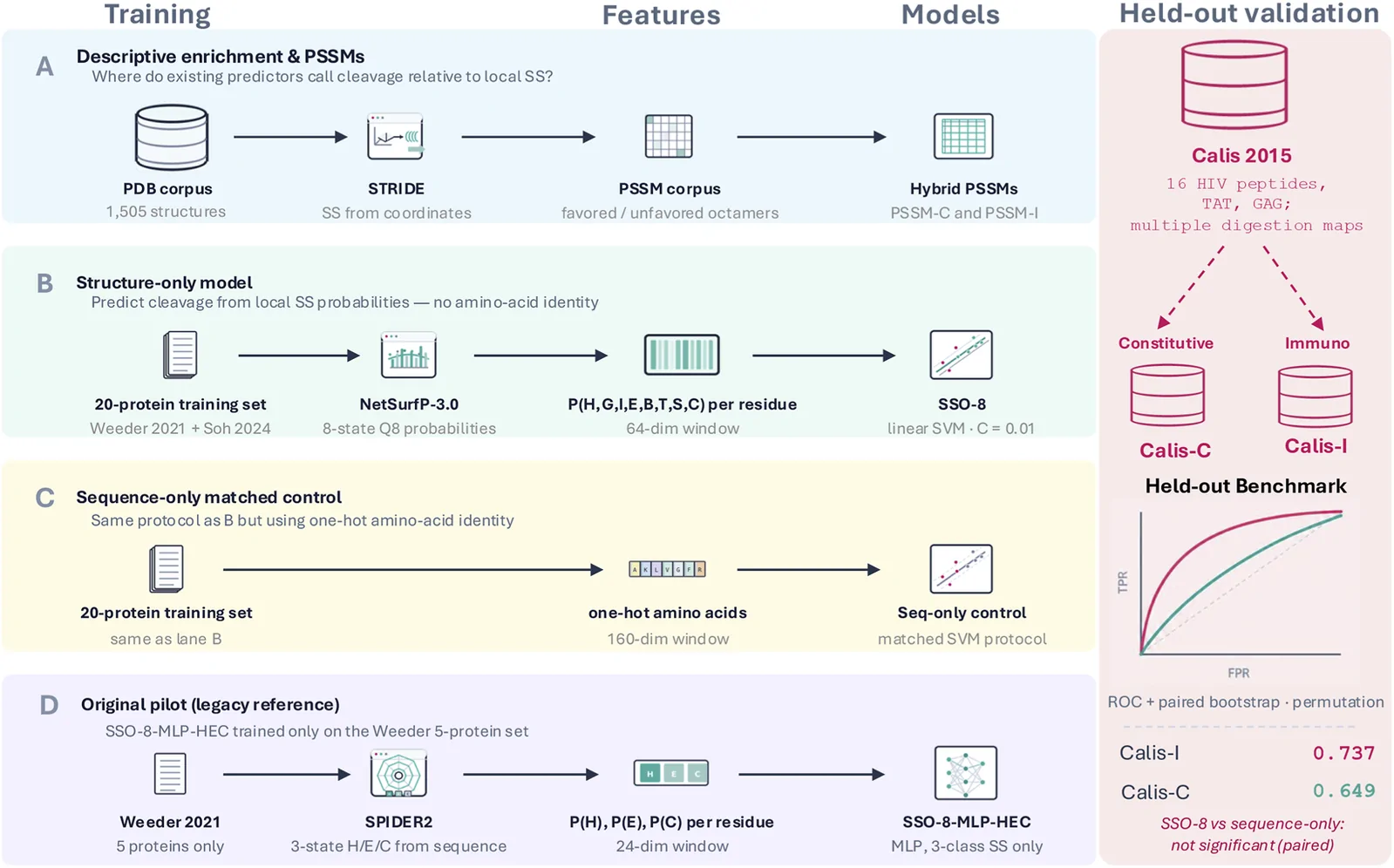
Workflow schematic for the analysis. Four analytical branches **(A–D)** converge on a common held-out benchmark (right). **(A)** Descriptive enrichment and PSSM construction. PDB corpus structures (1,505 proteins) were assigned secondary structure with STRIDE and scored with NetChop and ProteaSMM to build position-specific scoring matrices (PSSM-C and PSSM-I) from favored and unfavored octamer windows. **(B)** Structure-only model (SSO-8). The 20-protein experimental digestion dataset (Weeder 2021 and Soh 2024) was annotated with 8-state secondary-structure probabilities from NetSurfP-3.0, yielding 64-dimensional per-residue windows. A linear SVM (C = 0.01) was trained on these features without any amino-acid identity information. **(C)** Sequence-only matched control. The same 20-protein training set and window size were used, replacing secondary-structure probabilities with one-hot amino-acid encodings (160-dimensional), trained with the same SVM protocol as panel **(B)**. **(D)** Original pilot (legacy reference). The original SSO-8-MLP-HEC model, trained on the Weeder 2021 5-protein set using SPIDER2 3-state (H/E/C) secondary-structure probabilities in a 24-dimensional window and an MLP classifier, is retained for comparison. All branches were evaluated on the Calis 2015 held-out benchmark (16 HIV-derived peptides, TAT and GAG antigens, multiple digestion maps), split into constitutive (Calis-C, AUC = 0.649) and immunoproteasome (Calis-I, AUC = 0.737) benchmarks. Statistical comparison used ROC analysis with paired bootstrap and permutation tests.

For model training, we used a 20-protein experimental digestion dataset consisting of five proteins from (Weeder et al., 2021) and 15 proteins from (Soh et al., 2024) (Figure 2). The Soh 2024 dataset includes only human 20S constitutive proteasome cleavage patterns, while the Weeder 2021 includes both immunoproteasome and constitutive proteasome labels. Combined, this 20-protein dataset covered 1,215 experimentally-determined cleavage sites.

For secondary-structure enrichment in this dataset, we mapped the same substrates to available experimental PDB structures and to full-length predicted structures (Supplementary Table S1). The predicted-structure analysis covered all 20 proteins and all 1,215 cleavage sites. The experimental PDB analysis covered 17 proteins, as three intrinsically disordered proteins (alpha-synuclein, hTau, and beta-casein) lacked representative soluble experimental structures. Among the 1,060 cleavage sites in the non-IDP subset, 800 mapped to resolved PDB coordinates (75.5%).

To validate model performance, we used a separate benchmark dataset assembled by (Calis et al., 2015), which contains paired constitutive-proteasome (Calis-C) and immunoproteasome (Calis-I) cleavage annotations for 16 HIV-derived peptides. For the AUC calculations, terminal residues that cannot be assigned a downstream cleavage label were excluded. One sequence in the Calis 2015 validation dataset contained the non-standard residue code ‘X' (NAT10MOD). This residue was substituted with alanine for compatibility with predictors that require canonical amino-acid input.

### Sequence-based cleavage prediction and standard nomenclature

To assess whether secondary structure contributes predictive information beyond sequence, we compared our secondary-structure-only model (SSO-8) with established sequence-based proteasome predictors such as NetChop 20S, NetChop C-term, ProteaSMM-C and ProteaSMM-I (Tenzer et al., 2005; Nielsen et al., 2005), all of which were previously benchmarked on the Calis 2015 dataset.

Our references to cleavage positions follow the Schechter and Berger nomenclature that describes substrate-protease interactions. Amino acids where cleavage takes place are termed P1 at N-terminal residue and P1′ at the C-terminal residue (*e.g.*, P4, P3, P2, P1 |P1′, P2′, P3′, P4'…, where ‘|’ represents cleavage and the index increases for each amino acid N- or C-terminal). Substrate residues P4 to P1 interact with proteasome pockets S4 to S1, also called non-primed pockets, and residues P1′ to P4′ interact with substrate binding sites S1′ to S4′, or proteasome primed pockets (Harshbarger et al., 2015; Schechter et al., 1967).

### Secondary structure assignment and prediction

Secondary structure was extracted from PDBx/mmCIF coordinates using STRIDE (Heinig and Frishman, 2004), with labels mapped to three classes: helix (H, G, I), strand (E, B), and coil (C, T, blank, or other). Stride is explicitly designed to reproduce crystallographic secondary structure assignments (Heinig and Frishman, 2004), showing strong agreement with DSSP (Sladek et al., 2021), and comparable correlation to 3D structural similarity metrics such as TM-score and GDT-TS (Liu and Wang, 2018).

On the other hand, for sequence-based prediction of secondary-structures we used NetSurfP-3.0 (Høie et al., 2022), which provided us with per-residue eight-state secondary-structure probabilities for H, G, I, E, B, T, S, and C. These eight-state probabilities were used directly as features for SSO-8 model training, rather than collapsed into helix/strand/coil classes.

### Evaluation of secondary structure content in favored and unfavored cleavage sites

We first generated per-residue cleavage scores across the PDB corpus using NetChop C-term, NetChop 20S, ProteaSMM-I, and ProteaSMM-C. NetChop 3.1 was run as a standalone predictor, and ProteaSMM was implemented in Python (Van Rossum and Drake, 2011) from the published constitutive and immunoproteasome scoring matrices. ProteaSMM uses a 10-residue window to score cleavage between positions six and seven. Therefore, the first five and last four residues of each FASTA sequence were excluded from ProteaSMM-based analyses because a complete scoring window cannot be formed at those termini. For each predictor separately, residues were ranked by cleavage score. We selected the top 10% of scored residues as a prespecified high-confidence tail for secondary-structure enrichment analysis, rather than as an optimized biological cutoff. This group is referred to as favored predicted cleavage sites. Similarly, the bottom 10% of scored residues were retrieved as a low-confidence tail and are referred to as unfavored cleavage sites. Although the classification is made at the residue in position P1, subsequent analyses include local sequence windows (P6–P4’) around each site, effectively representing them as short peptide segments.

We then compared the secondary-structure composition at predicted cut sites against the overall PDB corpus background. First, we computed the “expected” frequencies of helices, β-strands (extended conformations), and coils across our entire PDB corpus dataset. We extended this analysis to positions P6–P4’ to evaluate how the observed/expected ratios fluctuate in the regions flanking cleavage sites. Although NetChop scores residues using a wider local window, we restricted this comparison to positions that could be evaluated across all methods.

### Structure-informed prediction of proteasomal cleavage

To evaluate the use of secondary structure data to support proteasome prediction we trained machine learning models using as input only secondary-structure probabilities. Our first implementation used secondary structure probabilities predicted by SPIDER2 (Yang et al., 2017) as the sole inputs to a feedforward neural network for cleavage prediction (*e*.*g*., using PyBrain (Schaul et al., 2010)). In that implementation, each amino acid was encoded by three neurons, representing its predicted probability of being a helix (H), coil (C), or (E) extended conformation (*i.e.*, three features per residue). Therefore, a 5-residue sequence required 15 input neurons (5 × 3), while a 12-residue sequence required 36 neurons (12 × 3). We employed a backpropagation algorithm with a momentum of 0.016, a learning rate of 0.06 and a validation of data proportion of 0.15. The output neuron was configured for binary classification, where a value of 1 represented a positive cleavage site and 0 represented a negative site. Various versions of the network using this 15-to-36 neuron input range were trained and the best performance was achieved with the 8-mer window. The optimized model was named SSO-8-MLP-HEC (Secondary Structure Only – 8 amino acids needed for prediction–Multi-Layer Perceptron using 3 secondary structure classes). Interestingly, an 8-mer window centered on the cleavage site can capture precisely the highly relevant biological positions from P4 to P4’ (Schechter et al., 1967). This early model was trained using only data from the small Weeder 2021 dataset and presented encouraging results on the Calis 2015 dataset (Supplementary Table S2).

The final SSO-8 model was trained on the expanded 20-protein training dataset (*i.e.*, Weeder 2021 + Soh 2024 substrates), while Calis-C and Calis-I were held out for final validation. Secondary-structure features were generated with NetSurfP-3.0. Each candidate cleavage site was represented by an 8-residue P4 to P4′ window containing eight secondary-structure probabilities per residue (64 features). Windows were aligned as P4-P1 on the N-terminal side and P1′-P4′ on the C-terminal side. Positions extending beyond a protein terminus were zero-padded so that all examples had the same 64-feature representation.

Constitutive and immunoproteasome labels were pooled from the 20-protein dataset for isoform-agnostic training. Candidate sites were defined as peptide bonds indexed by the residue immediately N-terminal to the scissile bond. A site was positive if it was cleaved by either isoform, and negative if the corresponding bond escaped cleavage across all available experimental conditions. Negative sites were therefore drawn from observed non-cleaved bonds in the mapped substrate sequences, rather than placed randomly. Sites with unavailable or ambiguous labels were excluded from training and evaluation.

Candidate model families included L2 logistic regression, elastic-net logistic regression, multilayer perceptrons, radial-basis-function support-vector machines, and linear support-vector machines, all evaluated with class balancing. Hyperparameters were selected only by protein-grouped cross-validation across the 20 training proteins. The locked SSO-8 model selected by this procedure was a linear support-vector machine (SVM) with C = 0.01. To test whether the SSO-8 model captured a secondary-structure signal rather than a generic local-window effect, we also trained a matched one-hot encoding sequence-only control using the same windows, labels, grouped cross-validation splits, and model-selection protocol.

### Hybrid PSSM construction

To complement single-predictor outputs with a consensus model, we constructed Position-Specific Scoring Matrices (PSSMs) from amino-acid octamers using a log-odds methodology. Octamers were 8-residue windows centered on each scoreable position in the PDB corpus, retaining the same window length used by SSO-8. Each window was labeled as “favored” or “unfavored” based on its rank in the score distribution of a predictor ensemble. Two main ensembles were used: SSO-8, NetChop 20S, and ProteaSMM-C for the constitutive PSSM (PSSM-C); and SSO-8, NetChop C-term, and ProteaSMM-I for the immunoproteasome PSSM (PSSM-I). Finally, we implemented a PSSM using the seq-only predictor (PSSM-seq-only) and another using both the seq-only and the SSO-8 predictor (PSSM-seq-n-SS), to isolate the baseline contribution of the sequence-only control and the additive contribution of SSO-8 to the ensemble.

Windows in the top 20% of ensemble scores formed the favored set, while those in the bottom 20% formed the unfavored set. This 20% tail, wider than the 10% used for the enrichment analyses, was chosen *a priori* to provide enough octamers for stable position-specific amino-acid frequency estimates, and was not tuned on the Calis validation benchmarks.

For each of the eight window positions, the observed amino-acid frequency *f*_*obs*_ (=*n*_*obs*_/*N*_*seq*_) was computed separately within the favored and unfavored sets and converted to a position-specific log-odds score, log (*F*_*obs*_/*F*_*exp*_), where *F*_*exp*_ was the background residue frequency across the full PDB corpus. This yielded two matrices per isoform: a favored PSSM profiling sequences with high cleavage probability and an unfavored PSSM profiling sequences with low cleavage probability. To score a candidate octamer, the log-odds entries from the favored matrix were summed and the corresponding sum from the unfavored matrix subtracted, yielding a single PSSM cleavage score. No Calis validation peptides contributed to PSSM construction, and the PSSMs were evaluated only on the held-out Calis-C and Calis-I benchmarks.

### Validation and statistical comparison of predictors

We assessed predictor performance using receiver operating characteristic (ROC) curves (Swets, 1988) and area under the ROC curve (AUC), computed with scikit-learn (Pedregosa et al., 2011). Confidence intervals were estimated by bootstrap resampling. Where per-residue scores were available for both predictors being compared, pairwise AUC differences were evaluated using paired resampling while preserving paired scores. For the SSO-8 versus sequence-only comparison, we treated Calis peptide/protein as the resampling unit and used both a paired bootstrap for AUC differences and a protein-level score-swap permutation test. We also performed protein-grouped cross-validation, checked for exact Calis peptide substrings in the PDB corpus, computed training-protein/PDB sequence identity, and used simplified two- and three-component PSSMs as sensitivity analyses for structure-aware hybrid predictors.

Dataset independence and potential sequence leakage were assessed through a four-part audit. Pairwise sequence identities were computed using global Needleman–Wunsch alignment (BioPython PairwiseAligner, match score = 1, mismatch = 0, gap open = −0.5, gap extend = −0.1), with 30% identity used as the standard homology-based independence threshold below which proteins are considered functionally unrelated. Three cross-set comparisons were performed: (i) the Weeder 2021 training subset (5 unique proteins) against the Soh 2024 training subset (15 proteins), yielding 75 pairs, to verify internal training independence; (ii) the merged 20-protein training set against all 16 Calis 2015 validation sequences, yielding 320 pairs, to verify training–validation independence; and (iii) the 20-protein training set against all 1,505 proteins of the PDB corpus used for PSSM construction, yielding 30,100 pairs, to characterize structural overlap between training proteins and the corpus. A fourth check addressed potential predictor-derived circularity: PSSM octamer weights are derived by labeling 8-residue windows in the PDB corpus as favored or unfavored based exclusively on the consensus of predicted scores from SSO-8, NetChop C-terminal, and ProteaSMM. No experimental cleavage annotation from any training or validation dataset enters the corpus labeling step.

## Results

### Favored cleavage sites are enriched with amino acids structured as β-strands

In the constitutive proteasome context, both NetChop 20S and ProteaSMM-C predict very similar observed/expected ratios (Figures 3A,C), with favored cleavage sites enriched in β-strand residues. Helices also show a modest enrichment, but the effect is weaker, more uniform across positions, and appears position-independent.

**FIGURE 3:**
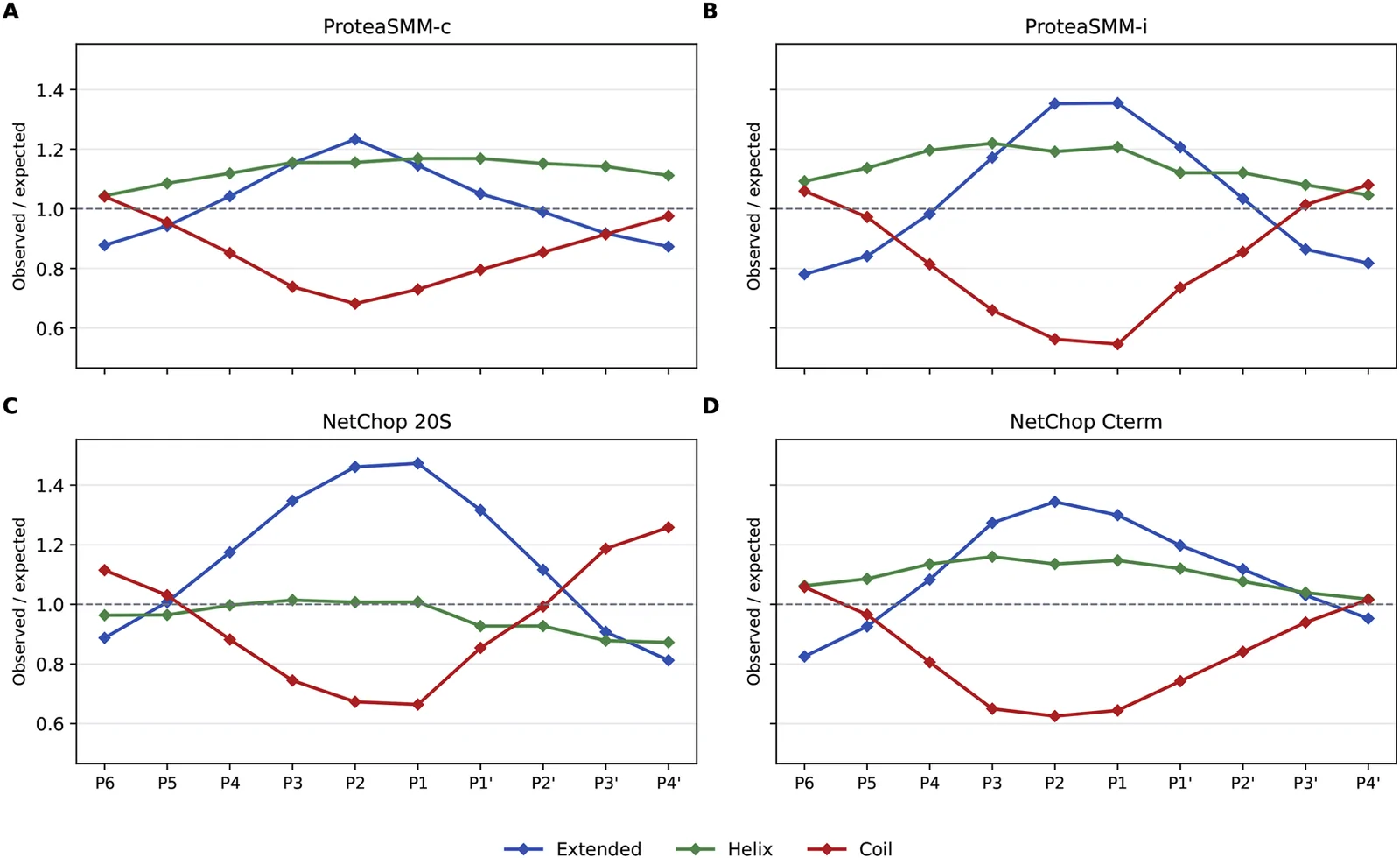
Favored predicted cleavage sites in the PDB corpus. Residues were sorted by predicted cleavage probability, and the observed/expected ratios of extended conformation (blue), helix (green), and coil (red) were plotted for the top 10% scored sites from P6 to P4’. Favored sites were predicted with ProteaSMM-C **(A)**, ProteaSMM-I **(B)**, NetChop 20S **(C)**, and NetChop C-term **(D)**.

For β-strands, the observed/expected ratios peak near the cleavage site and gradually decline with distance, forming a bell-shaped profile with fall off towards both the N and C termini. Coils are markedly underrepresented at these cut sites, mirroring the enrichment of β-strands in the same region. For ProteaSMM-C, position P2 is where we found the most prominent tendency to observe much more β-strands events. This same site is also where the observed/expected ratio of coils is minimal. NetChop 20S agrees that position P2 is the site where there are much more residues structured as extended conformation, and, together with P3 and P1, also present much fewer coils than expected by chance.

Using ProteaSMM-I or NetChop C-term (both of which incorporate immunoproteasome/MHC I-ligand data), we observe an even stronger enrichment of beta-strands at high-probability cleavage sites (Figures 3B,D). In the STRIDE-coordinate analysis, favored sites predicted by NetChop 20S were enriched for extended conformations (site-level E observed/expected = 1.473), and favored sites predicted by ProteaSMM-I were also enriched for extended conformations (E observed/expected = 1.355), with coils depleted in the corresponding favored-site sets. The exact magnitude depends on the PDB corpus and coordinate coverage, but the qualitative pattern is stable. This pattern can also be observed using either predicted secondary structure data (*e.g*., with NetSurfP-3.0) or crystallography-derived assignments (*e.g.*, with STRIDE), as shown in Supplementary Figure S1A.

### Neighboring regions of unfavored cleavage sites are enriched with coils

At unfavored cleavage sites, coils become enriched and both helices and beta-strands are underrepresented relative to background (Figures 4A–D). These results show that sequence-based cleavage scores are associated with local secondary-structure annotations, especially beta-strand enrichment and coil depletion. A direct true-label analysis of the 20-protein experimental digestion dataset showed the same tendency, with true cleavage sites being depleted for coils and enriched for helix and beta-strand assignments (Supplementary Figure S1B,C). This pattern was observed using both NetSurfP-3.0 predictions and STRIDE annotations. Differences in the magnitude of the enrichment in the Soh 2024 dataset in relation to the PDB corpus are likely related to its limited size (15 proteins), and cleavage patterns derived only from a constitutive proteasome. In addition, the PDB-structure subset of Soh 2024 has lower coverage due to unresolved regions in the crystallographic data, and the exclusion of disordered proteins (Supplementary Table S1).

**FIGURE 4:**
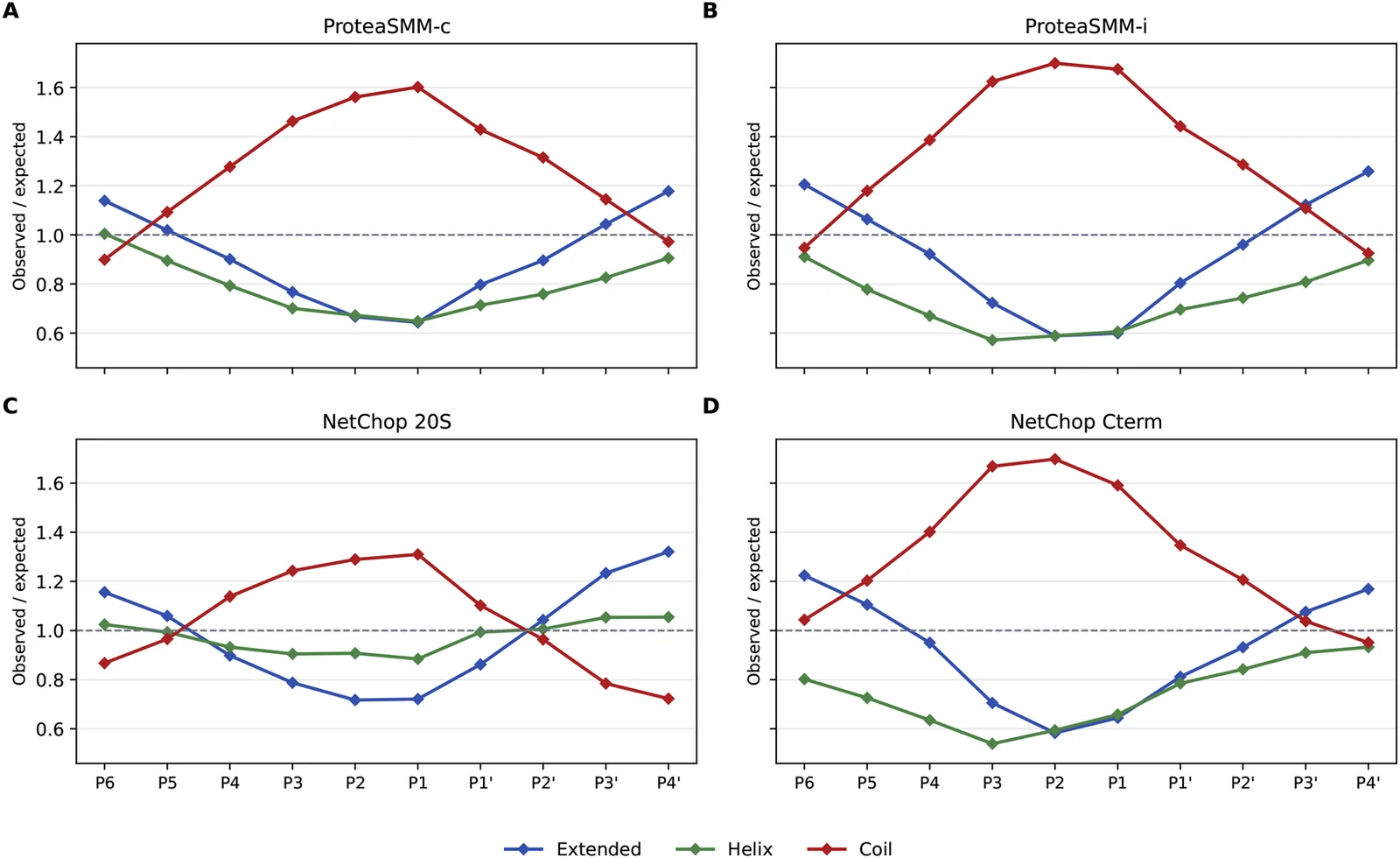
Unfavored predicted cleavage sites in the PDB corpus. Observed/expected ratios for extended conformation (blue), helix (green), and coil (red) were plotted for the bottom 10% scored sites from P6 to P4’. Unfavored sites were predicted with ProteaSMM-C **(A)**, ProteaSMM-I **(B)**, NetChop 20S **(C)**, and NetChop C-term **(D)**.

As an additional protein-level check, we compared mean predicted coil probability with mean NetChop C-term cleavage probability across proteins (Supplementary Figure S2). Coil content was inversely associated with predicted cleavage propensity in both the PDB corpus (r = −0.385, p = 2.15 × 10^−54^, n = 1,505 proteins) and the experimental digestion dataset (r = −0.736, p = 9.35 × 10^−5^, n = 22 digestion maps). This supports the same direction of association observed at the residue level, while controlling for artifacts of residue-level non-independence. Taken together, these analyses consistently show strong depletion of coils and enrichment of beta-strand and helix at cleavage sites, across diverse protein datasets, regardless of whether cleavage is defined by predictors or by experiment, and regardless of whether secondary structure is predicted or derived from crystallographic data.

### A reduced structural representation captures cleavage signal

We validated the SSO-8 model on held-out Calis constitutive and immunoproteasome cleavage benchmarks. The SSO-8 model reached AUC = 0.649 on Calis-C and 0.737 on Calis-I. The matched sequence-only control reached AUC = 0.674 and 0.736, respectively. Paired peptide/protein-level tests found no evidence of a significant SSO-8-vs-sequence difference on either benchmark (Supplementary Figure S3). The Calis-I comparison is especially striking because predicted secondary structure captured nearly the same cleavage signal as amino-acid identity while using less input information.

The SSO-8 method also displayed competitive performance relative to reference sequence-based methods in a benchmark using all scorable sites in the Calis dataset (Figure 5; Supplementary Figure S4; Supplementary Table S3). In the Calis-I dataset, SSO-8 reached AUC = 0.737, outperforming both NetChop 20S (AUC = 0.711) and NetChop C-term (AUC = 0.731). The sequence-only control model showed equivalent performance (AUC = 0.736).

**FIGURE 5:**
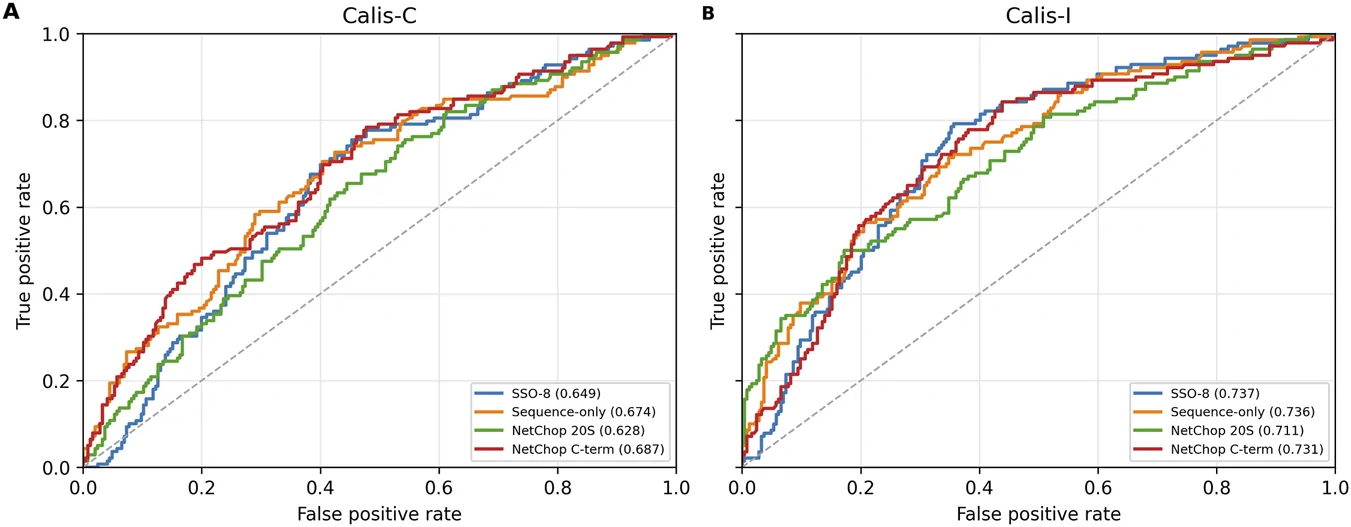
ROC curves for secondary-structure-based SSO-8 model, a sequence-only control model with matched architecture, and reference proteasomal cleavage models for constitutive (NetChop 20S) and immunoproteasome (NetChop C-term). Models were tested on Calis constitutive **(A)** and immunoproteasome **(B)** benchmarks. Complementary information also found in Supplementary Figure S4 and Supplementary Table S3.

The key observation is that secondary-structure alone, encoded as 64 eight-state probabilities across the P4–P4′ window, achieved competitive cleavage discrimination without any amino-acid identity information. This is a 2.5-fold reduction in input dimensionality relative to the 160-feature one-hot sequence encoding used by the matched control. In fact, when using a much smaller training dataset, a simpler three-class (H/E/C) secondary-structure encoding model (24-feature per 8-mer) outperformed the 160-feature sequence-only control on both the Calis-I (AUC = 0.726) and the Calis-C (AUC = 0.650) benchmarks (Supplementary Table S2). Together, these results indicate that local backbone conformation carries cleavage-relevant information, which might be particularly relevant on limited training datasets, and more strongly associated with immunoproteasome specificity.

We also performed protein-grouped cross-validation for the SSO-8 model on the 20-protein training dataset (Supplementary Figure S5). In the Leave One Protein Out (LOPO) experiment, SSO-8 had a grouped-CV mean AUC = 0.656, which is below its Calis-I performance (AUC = 0.737). Note that the matched sequence-only control showed the opposite pattern (LOPO mean = 0.812, Calis-I AUC = 0.736). The same inflation is observed for both PSSM variants on ProteaSMM-comparable sites (PSSM-seq-only: LOPO = 0.832, Calis-I = 0.781; PSSM-seq-n-SS: LOPO = 0.803, Calis-I = 0.797). This systematic inversion is consistent with local sequence patterns transferring within the 20-protein training set, which inflates LOPO performance for sequence-based models but does not affect SSO-8 predictions. We treat Calis-C and Calis-I as the primary held-out evidence, and grouped-CV as a check that model selection was not tuned directly to the validation peptides.

### PSSMs test additive effects of secondary structure data

We constructed hybrid PSSMs aggregating predictions from different methods, and this effort showed a small numerical increase when SSO-8 was added to sequence-based PSSM models, particularly on the immunoproteasome benchmark (Figure 6). This evaluation was performed in a subset of ProteaSMM-comparable residues, excluding terminal residues that cannot be scored by ProteaSMM (Supplementary Figures S6-S7; Supplementary Table S4).

**FIGURE 6:**
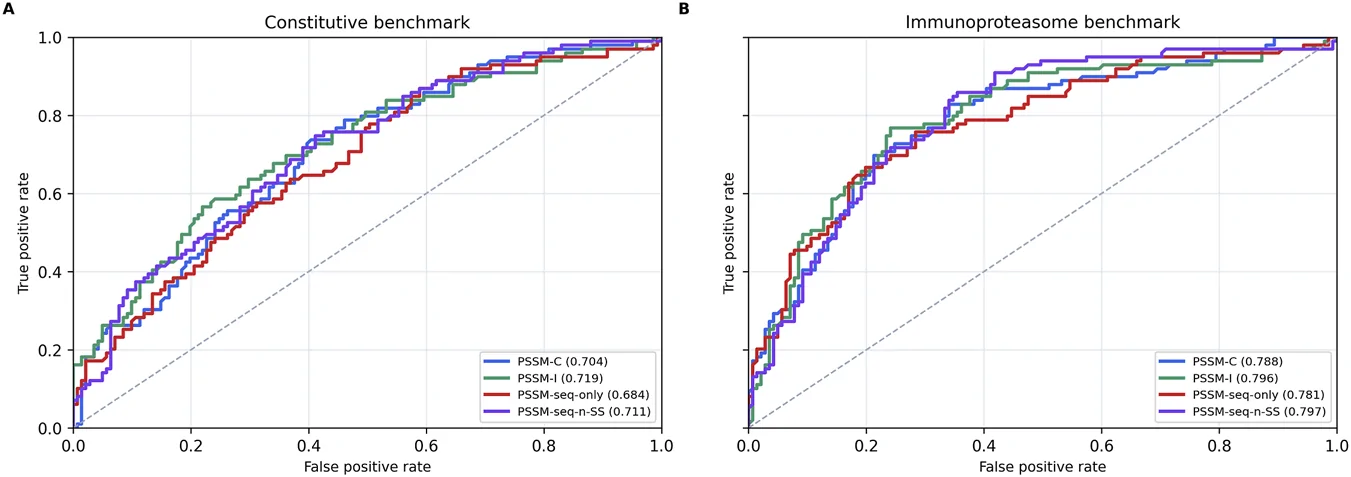
ROC curves for PSSM models on Calis constitutive **(A)** and immunoproteasome **(B)** benchmarks. PSSM-C uses SSO-8, NetChop 20S, and ProteaSMM-C; PSSM-I uses SSO-8, NetChop C-term, and ProteaSMM-I. PSSM-seq-only uses the matched sequence-only control, and PSSM-seq-n-SS pools sequence-only and secondary-structure-derived scores. Complementary information is provided in Supplementary Figures S6-S7 and Supplementary Table S4.

This contribution is made clearer when comparing PSSM-seq-only (AUC = 0.781) with PSSM-seq-n-SS (AUC = 0.797). This trend suggests that local substrate secondary structure could carry cleavage information that is partly orthogonal to sequence motifs. However, the AUC increase was not statistically significant by paired uncertainty analysis (bootstrap 95% CI for delta AUC = −0.027 to +0.057; paired permutation p = 0.30). The best PSSM performance, AUC = 0.806, was achieved with an ensemble including all five methods (Supplementary Table S4). The favored and unfavored thresholds used for enrichment and PSSM construction should be interpreted as prespecified tail definitions for a proof-of-concept analysis, not as optimized biological cutoffs.

The dataset independence audit confirmed the integrity of all cross-set boundaries. In the internal training comparison, no pair among the Weeder 2021 × Soh 2024 alignments exceeded 30% identity (maximum 28.7%, cEnolase vs. AnnexinA1), establishing that the two training subsets contribute non-redundant proteins. In the training–validation comparison, no pair among the 20-protein training × Calis 2015 alignments exceeded 30% identity (maximum 25.7%, HIV-1 Nef-1 vs. nat11); this maximum reflects an expected within-family relationship rather than data leakage. In the training–corpus comparison, 11 of 20 training proteins had at least one PDB corpus match above 30% identity (maximum 65.3%, UBE2S vs. 5BNB). This level of similarity is expected and does not constitute leakage because corpus octamer labels are assigned from predicted, not experimental, cleavage scores. Consequently, even where a training protein appears among the corpus entries, its experimental annotations cannot circularly re-enter the PSSM octamer weights. Taken together, the audit provided no evidence of sequence-level leakage between training and validation sets, and excludes predictor-derived circularity as a source of inflation in the reported performance estimates.

## Discussion

Here we show evidence that local substrate secondary structure is associated with proteasomal cleavage preferences and carries predictive information. The regions flanking the P1-P1’ cleavage sites are consistently depleted in coil-like annotations and enriched for ordered states, especially beta-strand or extended conformations. The reciprocal pattern is observed at unfavored sites, where coils were enriched and helices and beta-strands were underrepresented. The same patterns were observed across predicted and experimentally determined cleavage sites, and across predicted and structure-derived secondary-structure assignments, therefore suggesting that results reflect a true biological signal, and not an artificial methodological bias.

Our experiments with machine learning models extend this observation beyond the enrichment analysis. Despite using reduced input information that excluded amino-acid identities, our proof-of-concept secondary-structure-only models retained substantial cleavage-discriminatory information and were able to match or outperform sequence-only-control models using matched architecture and training dataset. Our results suggest that local backbone conformation carries cleavage-relevant information that may be especially useful when training data are limited.

Mechanistically, beta-strand enrichment may reflect several nonexclusive features of proteasomal processing. For instance, strand-like geometry may favor conformations that are more compatible with productive engagement of the proteasome catalytic site, whereas coils and turns may produce less favorable local geometry. Proteasome structures bound to peptide-like inhibitors and substrate analogues show a locally extended active-site conformation, with the reactive carbonyl or warhead positioned near the N-terminal catalytic threonine and the oxyanion-stabilizing environment (Tynd et al., 2005; Madala et al., 2010). The β-strands signal can therefore be reflective of compatibility with productive extended binding geometry, and not of direct selection for intact native β-strands before substrate engagement (Harshbarger et al., 2015; da Fonseca and Morris, 2015; Huber et al., 2016).

The observed signal also does not necessarily imply that intact native secondary structure survives translocation into the catalytic chamber (Ruschak et al., 2010; Berko et al., 2012). Instead, local sequence-encoded structural propensity may influence how a chain straightens, refolds, or packs during translocation (Ruschak et al., 2010; Prakash et al., 2004; Zhao et al., 2010; de la Peña et al., 2018). Future work could integrate molecular-dynamics ensembles (Conev et al., 2023) and downstream antigen-processing steps such as TAP transport (Tenzer et al., 2005), ERAP trimming (Evnouchidou and van Endert, 2019; Saulle et al., 2025; Fougiaxis et al., 2024), and peptide editing (van Hateren and Elliott, 2023) to further investigate the underlying mechanisms driving the observed secondary structure preferences.

Interestingly, this structural signal was not equally strong across proteasome isoforms. It was consistently more pronounced for immunoproteasome cleavage, particularly on the non-primed side of the cleavage site. This is important because amino-acid sequence partly determines local structural propensity, and therefore sequence-based predictors may implicitly learn secondary-structure preferences through sequence motifs rather than modeling residue-specific cleavage determinants alone. The hybrid PSSM results are consistent with that view. Structure-derived and sequence-derived scores were not interchangeable, and their combination performed best in the immunoproteasome benchmark. However, the modest size of the Calis-I validation set means the observed improvement remains statistically uncertain, and the PSSM result should be interpreted as directionally suggestive rather than conclusive.

To further investigate whether sequence-based cleavage predictors also capture this structural signal, we analyzed the relationship between predicted coil content and NetChop C-term cleavage probability. We found a consistent and statistically significant inverse correlation between NetChop C-term cleavage predictions and per-protein coil content in both the PDB corpus and the 20-protein experimental dataset (Supplementary Figure S2). Notably, NetChop C-term is a well-established sequence-based cleavage predictor trained on MHC class I ligand C-termini, with no explicit access to the secondary or tertiary structure of the original substrate proteins. Nevertheless, NetChop C-term predictions systematically disfavor residues in coil regions of the substrate, suggesting that the MHCI ligand training data implicitly encodes a secondary-structure preference that the model has captured through sequence motifs alone.

In addition, a *post hoc* analysis of the same DSSP-derived secondary-structure assignments used for Supplementary Figure S1 shows that beta-strand frequency at true immunoproteasome cleavage sites (32.5%) is significantly higher than at constitutive cleavage sites (18.7%; χ^2^ = 10.72, p = 0.001, Bonferroni-corrected), indicating that the two proteasome types carry distinguishable local structural signatures. These are interesting observations suggesting a role for substrate secondary structure in proteasomal cleavage, particularly for the immunoproteasome, which deserves further investigation. However, efficiently leveraging these findings to develop novel state-of-the-art (SOTA) proteasomal predictors remains an open challenge, that goes beyond the scope of the current study.

Although useful to support our proposed analysis, the SSO-8 model could not consistently outperform reference methods across different datasets. Beyond the reduced input representation used by SSO-8, this model was trained using isoform-agnostic pooling of constitutive and immunoproteasome cleavage sites, so that a single decision boundary had to accommodate both proteasome types. Considering the observed differences in secondary-structure preferences between isoforms, better predictive performance might be achieved by models that include proteasome type as an explicit feature. Unfortunately, training data remains limited for the intended application, particularly considering datasets containing both immunoproteasome digestion maps and protein structures. Here we explored the use of tools like STRIDE and NetSurfP-3.0 to predict secondary-structure labels for protein sequences, an approach that might be limiting our proteasomal prediction accuracy. In this context, Protein Language Models (PLMs) may offer an attractive alternative representation for future proteasomal cleavage models, since their embeddings encode information about structural propensity, evolutionary constraints, and long-range sequence context without requiring explicit secondary-structure prediction.

Additional limitations of our study include the fact that the PDB corpus used for some of the analysis consists of protein structures resolved at ≤2.5 Å resolution from the Protein Data Bank, which systematically under-represents intrinsically disordered regions and membrane proteins. The training datasets (*i.e.*, Weeder 2021; Soh 2024) were derived from *in vitro* reconstituted proteasome digestion assays, which lack the cellular context of chaperones, substrate unfolding machinery, and proteasome-associated regulators. The held-out validation dataset (Calis 2015) is limited to 16 HIV-derived peptides, and further generalization to larger datasets with immunoproteasome digestion maps remains untested. Finally, since we were focused on the potential contribution of secondary structure information, we did not incorporate training data from large-scale ligandome resources such as the Immune Epitope Database (IEDB) (Vita et al., 2025), which have been used by others to train immunoproteasome predictors (Amengual-Rigo and Guallar, 2021).

Since proteasomal cleavage produces the C-terminal anchor for peptide presentation by MHCI receptors, it provides a direct link between immunoproteasome specificity and epitope selection and presentation. We therefore hypothesize that the observed β-strand-associated pattern may bias the immunoproteasome’s peptide output toward structurally constrained regions of substrate proteins (Sitbon and Pietrokovski, 2007; Lesk et al., 1980), in turn favoring MHCI epitopes from regions where escape mutations are less tolerated (Kim et al., 2013). In this context, our results with SSO-8 and hybrid PSSMs suggest that future work should consider incorporating structural representations in the design of more accurate immunoproteasome prediction models, which could have broad biomedical implications for ongoing efforts in epitope/neoantigen discovery for vaccine development and T-cell-based immunotherapy design.

## Data Availability

Publicly available datasets were analyzed in this study. This data can be found here: Protein Data Bank: https://www.rcsb.org/. *In vitro* datasets used to train NetChop: https://services.healthtech.dtu.dk/services/NetChop-3.1/. Supplementary data on Soh et al., 2024: https://www.nature.com/articles/s41467-024-45339-3#Sec33.
