# Supplementary material for: Substrate secondary structure is associated with proteasomal cleavage propensity
Source: Front Bioinform. 2026 Oct 7;6:1730535. doi: 10.3389/fbinf.2026.1730535 (PMC13684360; doi:10.3389/fbinf.2026.1730535)
Supplement: Supplementary file 1 [file DataSheet1.pdf]

PDB IDs included in the PDB corpus (n = 1,505)

Row	PDB ID	PDB ID	PDB ID	PDB ID	PDB ID	PDB ID
1	1A12	2A8L	3AGY	4A8X	5A1H	8OHM
2	1A1X	2ABL	3AP5	4ABM	5A9B	
3	1A92	2AD1	3APM	4AE7	5A9C	
4	1AAP	2ALD	3AUV	4AE8	5AJS	
5	1AD6	2AUG	3AY5	4AGK	5ANO	
6	1AE9	2AW5	3B4D	4ANL	5ANR	
7	1AHS	2B2Y	3B7X	4ASZ	5AOO	
8	1AIE	2B3G	3B83	4B7L	5AQ5	
9	1AIL	2B49	3B93	4B8O	5AYS	
10	1AKZ	2B4I	3BAE	4B91	5BNB	
11	1ALY	2BK8	3BBC	4B93	5BXD	
12	1AOA	2BMJ	3BCH	4BCZ	5C10	
13	1ATZ	2BQ0	3BDW	4BD2	5C3T	
14	1AUE	2BSD	3BFO	4BD6	5C5S	
15	1AVY	2BT2	3BH0	4BG7	5C6K	
16	1AX8	2BTZ	3BIN	4BKG	5CEN	
17	1B34	2BZV	3BKJ	4BL7	5CFC	
18	1B35	2BZY	3BOR	4BOU	5CFD	
19	1B3U	2C0Y	3BQO	4BQQ	5CJ0	
20	1B4F	2C3T	3BQQ	4BT9	5CJ1	
21	1B77	2C46	3BU8	4BWN	5CRU	
22	1BAZ	2C6U	3BUA	4BWQ	5CS1	
23	1BBC	2CAR	3BVP	4BWS	5CV0	
24	1BCO	2CB5	3BYI	4C0F	5CZ1	
25	1BD8	2CE8	3C5R	4C41	5D5K	
26	1BD9	2CE9	3C5V	4C5Q	5DCK	
27	1BFG	2CG7	3C8X	4CA0	5DVL	
28	1BHD	2CJR	3CAA	4CAY	5DWP	
29	1BHS	2CKW	3CAF	4CDO	5DZM	
30	1BIS	2CL3	3CC0	4CEK	5E0U	
31	1BKL	2CLR	3CEY	4CHD	5E0V	
32	1BKR	2CMP	3CI9	4CHE	5E5B	
33	1BKZ	2CRO	3CJW	4CPC	5E5X	
34	1BP5	2D07	3CO1	4CRH	5E5Z	
35	1BR9	2D0J	3COO	4CT6	5E7I	
36	1BRQ	2D5R	3COU	4CU2	5E8S	
37	1BUO	2D68	3CSR	4CU5	5EAY	
38	1BY7	2DOK	3CTB	4CVD	5EF9	
39	1BY9	2DSB	3CVM	4CXI	5EFS	

Row	PDB ID	PDB ID	PDB ID	PDB ID	PDB ID	PDB ID
40	1BZ4	2DWY	3CX2	4CY2	5EFX	
41	1C25	2DYL	3D0V	4D5R	5EI0	
42	1C48	2DYP	3D24	4D8O	5EIQ	
43	1C5M	2E0J	3D3K	4D9O	5EKF	
44	1CA4	2E0T	3D3M	4DDP	5ENA	
45	1CA9	2E3H	3D7C	4DGJ	5ET5	
46	1CE0	2E3I	3D95	4DJ9	5F18	
47	1CJD	2E3S	3D9H	4DOT	5F3K	
48	1CN3	2E5B	3DA0	4DQ7	5F7F	
49	1CQ3	2E7A	3DAB	4DYL	5FGC	
50	1CSK	2EB2	3DAC	4DYQ	5FMO	
51	1CZT	2ED6	3DAD	4E1I	5HDW	
52	1D4T	2EDM	3DAL	4E9E	5HFQ	
53	1DDJ	2EJ8	3DD9	4ECB	5HIH	
54	1DEU	2EK6	3DG1	4EEW	5HPG	
55	1DEV	2ELA	3DGS	4EF0	5HPS	
56	1DF4	2END	3DJ9	4EF5	5HQG	
57	1DG3	2ERF	3DJU	4EFO	5HUW	
58	1DLE	2ES0	3DKB	4EGT	5HVZ	
59	1DOL	2ETX	3DKM	4EJQ	5HX4	
60	1DPT	2EXE	3DLM	4EJR	5I62	
61	1DSU	2F15	3DRE	4ELL	5I8C	
62	1DTJ	2F1S	3DRZ	4EO0	5IBK	
63	1DUN	2F4W	3DSH	4ERN	5IBM	
64	1DYN	2F5J	3DXE	4EYC	5IO9	
65	1E6F	2F5K	3DXF	4EZA	5IP4	
66	1E8Y	2F5U	3E0C	4F11	5IY3	
67	1EDN	2F73	3EAB	4F25	5IZH	
68	1EE4	2FAZ	3EAE	4F3J	5J09	
69	1EG3	2FCB	3EAZ	4F59	5J7E	
70	1EG4	2FCF	3EBN	4F80	5J8E	
71	1EI7	2FH7	3EDU	4FAE	5J8R	
72	1EJO	2FHT	3EGN	4FBN	5JD0	
73	1EL6	2FJR	3EJF	4FDD	5JEM	
74	1EOG	2FJZ	3EJG	4FHQ	5JMT	
75	1EVS	2FK9	3ELP	4FI5		
76	1F05	2FKL	3ENB	4FIT		
77	1F13	2FOJ	3EP0	4FQN		
78	1F14	2FOO	3EQ5	4FW1		
79	1F2L	2FOP	3ERB	4G03		
80	1F39	2FQM	3ETI	4G1O		
81	1F3U	2FRG	3EWQ	4G3O		

Row	PDB ID	PDB ID	PDB ID	PDB ID	PDB ID	PDB ID
82	1F6W	2FSD	3EXV	4G6A		
83	1F7O	2FXO	3EY6	4G6F		
84	1FEW	2FY2	3F02	4G8K		
85	1FGU	2FYZ	3F1I	4G94		
86	1FM5	2FZP	3F1P	4GDO		
87	1FNA	2G3T	3F2U	4GEI		
88	1FNF	2G69	3F31	4GGA		
89	1FP5	2G7I	3F62	4GGD		
90	1FQN	2GEC	3FAU	4GLI		
91	1FS1	2GEE	3FD4	4GMV		
92	1FVR	2GF2	3FDR	4GVB		
93	1FZR	2GGV	3FDW	4GWR		
94	1G1C	2GHV	3FE3	4H39		
95	1G1U	2GIY	3FFL	4H3B		
96	1G2C	2GMF	3FFM	4H5S		
97	1G40	2GPH	3FG7	4H7Y		
98	1G8Q	2GPO	3FKC	4H9J		
99	1GH2	2GRC	3FKE	4HBE		
100	1GIF	2GRR	3FLG	4HJP		
101	1GK6	2GTG	3FPO	4HLX		
102	1GLO	2GTR	3FRR	4HPL		
103	1GO4	2GZV	3FSO	4HQA		
104	1GS9	2H2C	3FTK	4HR9		
105	1GSM	2H2R	3FTL	4HRG		
106	1GVJ	2H2Z	3FTR	4HS2		
107	1GVL	2H3L	3FXY	4HS6		
108	1GY5	2H3N	3FZO	4HT1		
109	1GZK	2H4U	3G03	4HTJ		
110	1H03	2H6D	3G1G	4HWN		
111	1H09	2H6P	3G29	4HXJ		
112	1H4A	2HDZ	3G5Y	4HZA		
113	1H59	2HE2	3G80	4I6X		
114	1H6W	2HE7	3GAX	4ICU		
115	1HA4	2HKF	3GG6	4ICW		
116	1HAR	2HNT	3GLK	4IDH		
117	1HCG	2HQX	3GPG	4IFL		
118	1HDM	2HZF	3GQH	4IL7		
119	1HHI	2I0L	3GSZ	4IPC		
120	1HHJ	2I3U	3GV3	4IQH		
121	1HHK	2I6L	3GW3	4IQM		
122	1HKF	2IBL	3H00	4IRG		
123	1HOC	2IC6	3H0I	4IUJ		

Row	PDB ID	PDB ID	PDB ID	PDB ID	PDB ID	PDB ID
124	1HPT	2IC9	3H13	4IV1		
125	1HTR	2ICC	3H8K	4IYS		
126	1HUC	2II0	3H8M	4J0U		
127	1HUL	2IJ0	3HAK	4J0W		
128	1HUW	2ILR	3HBW	4J4R		
129	1I2T	2IPR	3HC7	4J4Y		
130	1I7K	2IU1	3HD4	4J5Q		
131	1IAP	2IUG	3HDE	4J6P		
132	1IED	2IUM	3HFE	4J77		
133	1IHB	2IWN	3HI9	4J79		
134	1IJB	2IWO	3HJ2	4J81		
135	1IJQ	2IWQ	3HME	4J82		
136	1ILK	2IXM	3HMH	4J8S		
137	1ILR	2J2J	3HMT	4J8Z		
138	1IMF	2J4O	3HNY	4JHN		
139	1IU1	2J67	3HRN	4JHP		
140	1J1J	2J6B	3HRO	4JIF		
141	1J2F	2J6F	3HTU	4JJQ		
142	1J72	2J6O	3HYD	4JKQ		
143	1JIQ	2J8B	3I00	4JMI		
144	1JJH	2J92	3I0C	4JMQ		
145	1JKG	2JA4	3I2E	4JNU		
146	1JKS	2JDL	3I2N	4JOI		
147	1JPS	2JDQ	3I35	4JOP		
148	1JPW	2JDW	3I6X	4JPN		
149	1JQ0	2JEO	3I85	4JPP		
150	1JR2	2JEX	3I8Z	4JQ5		
151	1JSG	2JGC	3IFL	4JQF		
152	1JUO	2JGN	3IFN	4JU5		
153	1JWF	2JGY	3IFO	4JZ5		
154	1K1B	2JIK	3IHX	4JZC		
155	1K95	2JJ6	3IK8	4JZQ		
156	1KAF	2JJW	3IKK	4K2O		
157	1KEX	2JK9	3INK	4K7E		
158	1KFU	2MHA	3IU5	4K7F		
159	1KKU	2NLK	3IVV	4K8U		
160	1KMT	2NLS	3IWP	4KG8		
161	1KNB	2NML	3IX0	4KG9		
162	1KPB	2NN2	3JQH	4KI5		
163	1KW2	2NPT	3JSB	4KY9		
164	1KXF	2NWD	3JXF	4L00		
165	1L2H	2NZ7	3K51	4L0N		

Row	PDB ID	PDB ID	PDB ID	PDB ID	PDB ID	PDB ID
166	1L3K	2O0K	3K9A	4L0R		
167	1LCL	2O2K	3KB5	4L1D		
168	1LCY	2O2V	3KCI	4L2W		
169	1LGQ	2O4X	3KN6	4L4R		
170	1LIT	2O71	3KT2	4L5F		
171	1LM5	2OB4	3KT9	4LD8		
172	1LTO	2OBI	3KXS	4LDM		
173	1M0Z	2OC3	3KZ3	4LDR		
174	1M1E	2OCT	3KZD	4LHF		
175	1M5I	2ODU	3L32	4LLA		
176	1MBM	2OEI	3LCY	4LLD		
177	1MF7	2OG3	3LD1	4LLI		
178	1MHN	2OGQ	3LDN	4LN2		
179	1MIZ	2OKR	3LE4	4LNP		
180	1MJS	2OMQ	3LJB	4LNR		
181	1MK7	2ON9	3LK4	4M4Z		
182	1MKF	2ONO	3LKX	4M67		
183	1MML	2OPW	3LOZ	4M7C		
184	1MN8	2OSA	3LQ6	4M91		
185	1MSC	2OT3	3LRU	4M92		
186	1MUK	2OU1	3LSD	4M9K		
187	1MZA	2OUC	3LWE	4M9P		
188	1MZW	2OUJ	3M5B	4MJJ		
189	1N1J	2OVA	3M5R	4ML7		
190	1N7F	2OVC	3M66	4MOD		
191	1N93	2P1L	3MAC	4MQ3		
192	1NA7	2P23	3MDF	4MQV		
193	1NCF	2P3W	3MEW	4MZ2		
194	1NF1	2P84	3MFT	4MZ5		
195	1NFN	2P8Q	3MGM	4MZ6		
196	1NG2	2P9R	3MGO	4N1D		
197	1NI2	2PB7	3MGT	4N1L		
198	1NKO	2PE6	3MJK	4N3X		
199	1NKR	2PE8	3MK4	4N3Y		
200	1NM8	2PEH	3MLR	4N4A		
201	1NO4	2PET	3MLV	4N6L		
202	1NOZ	2PGG	3MLY	4N7F		
203	1NR2	2PN8	3MOQ	4N8V		
204	1NRV	2PO4	3MP4	4NAC		
205	1NTY	2PPI	3MQP	4NCU		
206	1O3X	2PPN	3MQR	4NFB		
207	1O7Z	2PQ5	3MQS	4NIO		

Row	PDB ID	PDB ID	PDB ID	PDB ID	PDB ID	PDB ID
208	1OAI	2PQA	3MTS	4NIP		
209	1OEM	2PZ1	3MX7	4NJ8		
210	1OHC	2Q13	3MYI	4NK0		
211	1OIA	2Q20	3N3F	4NKJ		
212	1OLZ	2Q5X	3NAP	4NLH		
213	1OM9	2Q6D	3NDD	4NP8		
214	1ORC	2Q87	3NFN	4NPN		
215	1OW1	2Q8R	3NFY	4NRX		
216	1OW6	2Q8T	3NR5	4NSP		
217	1OX3	2Q9Q	3NRX	4NSW		
218	1P27	2Q9V	3NSO	4NUU		
219	1P32	2QBW	3NXA	4NUX		
220	1P4U	2QCQ	3O5E	4O00		
221	1P6F	2QCW	3O5P	4O1V		
222	1P7S	2QOL	3O6L	4O2E		
223	1PBW	2QPW	3O6M	4O2F		
224	1PCF	2QQ5	3O8V	4O6X		
225	1PJM	2QY7	3OBQ	4O7Q		
226	1PRG	2QYP	3OBU	4OJF		
227	1Q1T	2R18	3OBX	4OM7		
228	1Q2H	2R3V	3OHU	4ONF		
229	1Q5U	2R55	3OLN	4ONL		
230	1Q7S	2RB8	3ON9	4OO4		
231	1Q94	2RBH	3OQS	4OS3		
232	1QB2	2RCZ	3OU5	4OSN		
233	1QGK	2REA	3OY2	4OUH		
234	1QGR	2REY	3P4F	4OV5		
235	1QGV	2RFJ	3P4L	4P09		
236	1QIU	2RJD	3P54	4P0A		
237	1QMT	2RJL	3P8D	4P0L		
238	1QNT	2RK3	3P9A	4P2K		
239	1QQG	2RL0	3PBH	4P39		
240	1QQH	2RSP	3PD7	4PA1		
241	1QR1	2TGI	3PH9	4PAS		
242	1QU1	2UUZ	3PT3	4PH1		
243	1QUQ	2UWI	3PTH	4PQD		
244	1QUU	2V0V	3Q0H	4PR5		
245	1QVO	2V17	3Q6L	4PRA		
246	1QX3	2V5F	3Q6S	4PRE		
247	1QZQ	2VC8	3Q90	4PRH		
248	1R1W	2VFY	3Q9P	4PSF		
249	1R29	2VGA	3QC7	4PWQ		

Row	PDB ID	PDB ID	PDB ID	PDB ID	PDB ID	PDB ID
250	1R2D	2VGE	3QC8	4Q2O		
251	1R4B	2VH7	3QCN	4Q2Q		
252	1R69	2VKT	3QF2	4Q3H		
253	1R6K	2VKY	3QIR	4QB0		
254	1R9W	2VL3	3QR2	4QDR		
255	1RAJ	2VPH	3QSQ	4QFT		
256	1RCB	2VPI	3QWM	4QGK		
257	1REG	2VPK	3QXL	4QMD		
258	1RGP	2VR3	3QYE	4QMI		
259	1RHG	2VSP	3R2P	4QVA		
260	1RNF	2VSV	3R3P	4QXX		
261	1RPM	2VSW	3R7G	4R0B		
262	1RU7	2VSZ	3R7S	4R0P		
263	1S4Y	2VTY	3R8Q	4R0U		
264	1S9H	2VVW	3RD2	4R0W		
265	1SC4	2VWF	3REA	4R7V		
266	1SGK	2VWR	3RJ2	4R8R		
267	1SGZ	2VX2	3RK6	4RDJ		
268	1SH0	2VXP	3RKC	4RIK		
269	1SNT	2VXW	3RLN	4RIS		
270	1SNZ	2VY6	3RNV	4RM8		
271	1SQW	2VY8	3RPP	4RP6		
272	1STM	2VYI	3RRQ	4RPD		
273	1SYQ	2W0G	3RVC	4RSV		
274	1T3Y	2W0I	3RWC	4RT4		
275	1T45	2W0Z	3RWI	4RUG		
276	1T5I	2W5H	3RZ9	4RXH		
277	1T5R	2W80	3RZY	4RZ9		
278	1T6F	2W8M	3S9G	4RZA		
279	1T6L	2W9Z	3SD4	4S0U		
280	1T6O	2WA0	3SGS	4TLQ		
281	1T77	2WB8	3SH4	4TSE		
282	1T7H	2WL8	3SHT	4TUJ		
283	1T94	2WLV	3SKM	4TXQ		
284	1TCZ	2WQI	3SQF	4U0C		
285	1TD0	2WQJ	3SWY	4U2M		
286	1TEN	2WUX	3T0H	4U4H		
287	1TFC	2WWE	3T1W	4U4P		
288	1TKI	2WWX	3T30	4UED		
289	1TOL	2WX3	3T3K	4UEX		
290	1TUL	2WZ9	3T5X	4ULW		
291	1TVD	2X35	3TCQ	4UMI		

Row	PDB ID	PDB ID	PDB ID	PDB ID	PDB ID	PDB ID
292	1TXD	2X36	3TF3	4UML		
293	1TZS	2X3M	3TF5	4UNL		
294	1U2H	2X4I	3TG7	4UUC		
295	1U7B	2X4J	3TOJ	4UYI		
296	1UCH	2X5Y	3TOW	4V0P		
297	1UCT	2X6M	3TQ7	4V0T		
298	1UFI	2X78	3TSV	4V1P		
299	1UFQ	2X9A	3TSZ	4V2B		
300	1UG3	2XD7	3TUO	4V2D		
301	1UGN	2XF7	3TV0	4W5X		
302	1UII	2XSS	3TW8	4WAU		
303	1ULH	2XTC	3TXS	4WBE		
304	1UNP	2XXN	3TYY	4WFT		
305	1UOH	2XZZ	3U0R	4WHT		
306	1US7	2Y29	3U0T	4WJO		
307	1USE	2Y3J	3U0V	4WJQ		
308	1UTC	2Y3K	3U30	4WLR		
309	1UTY	2Y3L	3U3I	4WTX		
310	1UZ3	2Y7L	3U3P	4WW1		
311	1UZQ	2Y8O	3U7D	4WWR		
312	1V05	2YD5	3U84	4WY4		
313	1V1H	2YDL	3UH8	4X1M		
314	1VCA	2YGS	3ULL	4XAL		
315	1VCC	2YHF	3ULR	4XDX		
316	1VHI	2YLE	3UMN	4XEF		
317	1VKA	2YPK	3UMZ	4XEK		
318	1VLK	2YQ1	3UR6	4XEV		
319	1VPN	2YVQ	3UUN	4XFQ		
320	1VQB	2YXM	3UVL	4XH3		
321	1VSM	2YY0	3UVM	4XH9		
322	1W24	2YYN	3UVN	4XHT		
323	1W4C	2Z0W	3UVO	4XOF		
324	1W4V	2Z14	3V1Q	4XW3		
325	1W6X	2Z15	3V30	4XXD		
326	1W7B	2Z16	3V32	4XZA		
327	1WER	2Z5E	3V33	4XZG		
328	1WKU	2Z5S	3V42	4Y6X		
329	1WLZ	2Z5T	3V55	4YE6		
330	1WM3	2Z6H	3V5D	4YFW		
331	1WOU	2ZBS	3V6L	4YKD		
332	1WP8	2ZFH	3V9G	4YNO		
333	1WPA	2ZFY	3VC8	4YON		

Row	PDB ID	PDB ID	PDB ID	PDB ID	PDB ID	PDB ID
334	1WRU	2ZNL	3VFI	4YPC		
335	1WT6	2ZQQ	3VIA	4YUD		
336	1WU9	2ZU1	3VJ8	4YUO		
337	1WVN		3VOQ	4YX7		
338	1WWB		3VVV	4Z2N		
339	1WWC		3WFV	4Z8E		
340	1X8Y		3WI0	4Z8J		
341	1XAK		3WOY	4Z99		
342	1XAW		3WPQ	4Z9P		
343	1XBA		3WTD	4ZAI		
344	1XBS		3WVZ	4ZAJ		
345	1XGD		3WX4	4ZBW		
346	1XGW		3X11	4ZC3		
347	1XKS		3X12	4ZCB		
348	1XO1		3X13	4ZIF		
349	1XQZ		3X14	4ZOU		
350	1XWG		3X23	4ZPC		
351	1YCK		3ZBD	4ZQS		
352	1YDE		3ZDL	4ZTI		
353	1YDI		3ZHI	4ZTO		
354	1YGS		3ZSL	4ZUU		
355	1YH2		3ZXP	4ZUV		
356	1YHV		3ZZY	4ZVX		
357	1YIB			4ZWQ		
358	1YOJ					
359	1YPU					
360	1YPY					
361	1YQB					
362	1YRV					
363	1YTQ					
364	1YWO					
365	1YYH					
366	1YZ1					
367	1YZM					
368	1Z7G					
369	1Z9M					
370	1ZD8					
371	1ZJH					
372	1ZJK					
373	1ZLM					
374	1ZM0					
375	1ZMM					

Row	PDB ID	PDB ID	PDB ID	PDB ID	PDB ID	PDB ID
376	1ZON					
377	1ZV4					
378	1ZVA					
379	1ZVB					
380	1ZXT					
381	1ZZK					
