# Supplementary material for: Substrate secondary structure is associated with proteasomal cleavage propensity
Source: Front Bioinform. 2026 Oct 7;6:1730535. doi: 10.3389/fbinf.2026.1730535 (PMC13684360; doi:10.3389/fbinf.2026.1730535)
Supplement: Supplementary file 2 [file DataSheet2.pdf]

SUPPLEMENTARY MATERIAL

Substrate Secondary Structure Is Associated with Proteasomal Cleavage Propensity

Matheus de Bastos Balbé e Gutierres[1†], Finn Beruldsen[2†], Dinler Amaral Antunes[2*], Gustavo Fioravanti Vieira [1,3*]

[1] Programa de Pós-Graduação em Genética e Biologia Molecular (PPGBM-UFRGS), Porto Alegre, Brazil.

[2] Department of Biology and Biochemistry, University of Houston, USA.

[3] Programa de Pós-Graduação em Saúde e Desenvolvimento Humano – PPGSAUDEH, Universidade La Salle, Canoas, Brazil.

† These authors contributed equally to this work.

*** Correspondence:**

Gustavo Fioravanti Vieira, PhD (gustavo.vieira@unilasalle.edu.br);

Dinler Amaral Antunes, DSc (dinler@uh.edu).

SUPPLEMENTARY MATERIAL

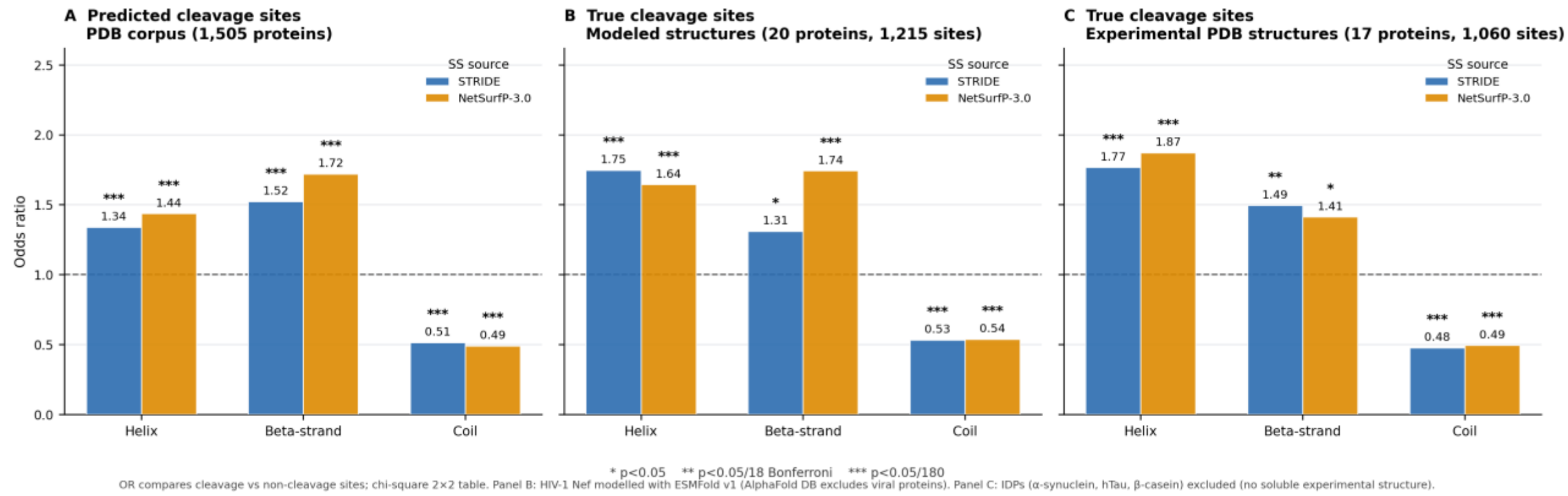

Supplementary Figure S1. Secondary-structure enrichment at predicted and true proteasomal cleavage sites. Odds ratios (OR) comparing the frequency of each secondary-structure class (Helix, Beta-strand, Coil) between cleavage and non-cleavage sites. Two independent secondary-structure sources are shown: STRIDE (coordinate-based, dark blue) and NetSurfP-3.0 (sequence-based, orange). ORs were computed from 2×2 contingency tables; Haldane–Anscombe correction (+0.5 to all cells) was applied when any cell was zero. (A) Predicted cleavage sites in the PDB corpus (1,505 proteins), pooled across four predictors (NetChop 20S, NetChop C-term, ProteaSMM-C, ProteaSMM-I). (B) True cleavage sites mapped to predicted structures for the 20-protein experimental digestion dataset (1,215 sites). (C) True cleavage sites mapped to experimental PDB structures for the non-IDP subset (17 proteins; 1,060 sites). Significance was assessed by chi-square test with Bonferroni correction for 18 simultaneous comparisons (3 panels × 2 SS sources × 3 classes): *$p < 0.05$; **$p < 0.05/18$; ***$p < 0.05/180$. Structure coverage is summarized in Supplementary Table S1.

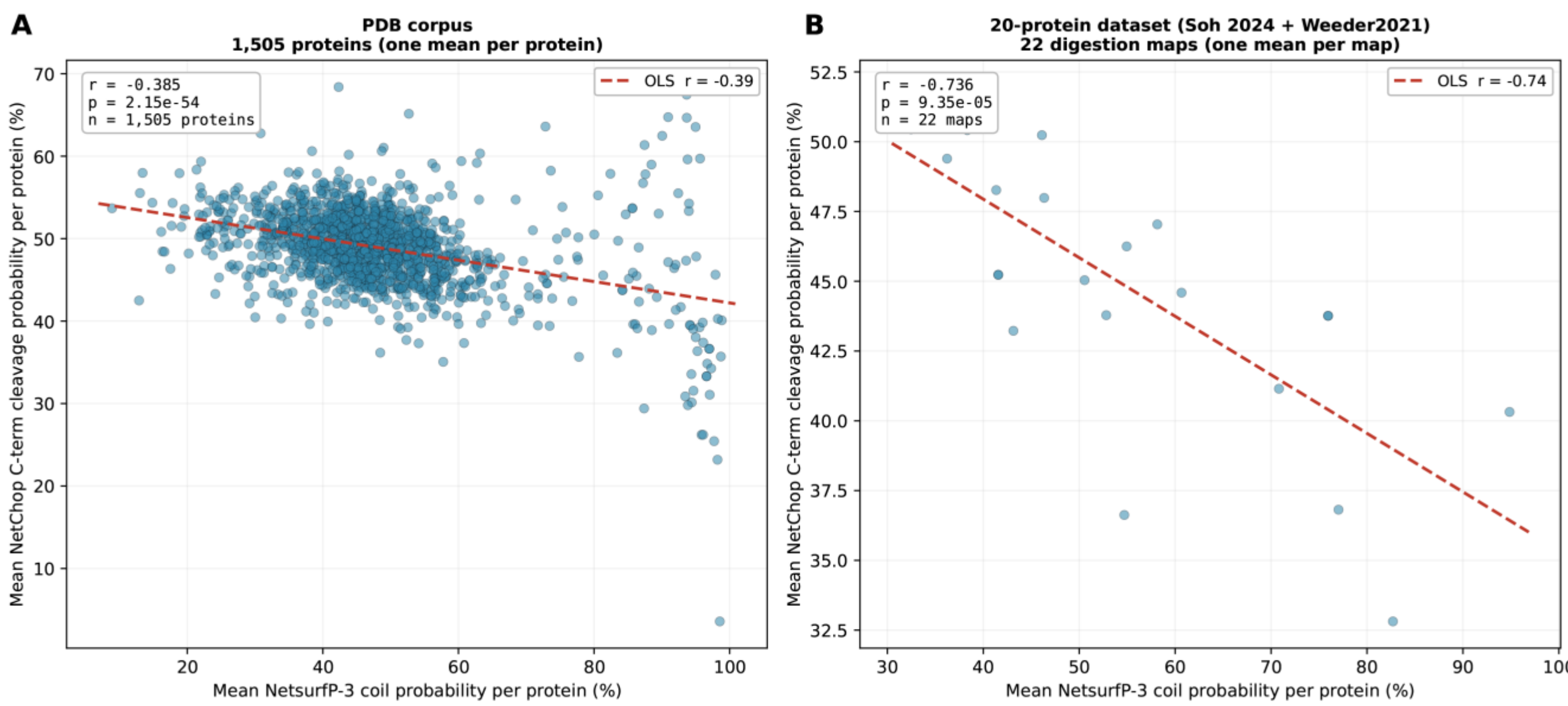

Supplementary Figure S2. Protein-level association between predicted coil content and immunoproteasome cleavage probability. Each point represents one protein (A) or one digestion map (B), plotted as the mean NetsurfP-3.0 coil probability against the mean NetChop C-term cleavage probability. (A) PDB corpus (1,505 proteins): Pearson $r = -0.385$, $p = 2.15\times10^{-54}$. (B) 20-protein experimental digestion dataset (Soh 2024 + Weeder 2021; 22 digestion maps): Pearson $r = -0.736$, $p = 9.35\times10^{-5}$. Dashed red lines show ordinary least squares (OLS) regression fits. The inverse correlation is statistically significant at the protein level in both datasets, confirming that the association between substrate secondary structure and proteasomal cleavage propensity is not an artifact of residue-level non-independence.

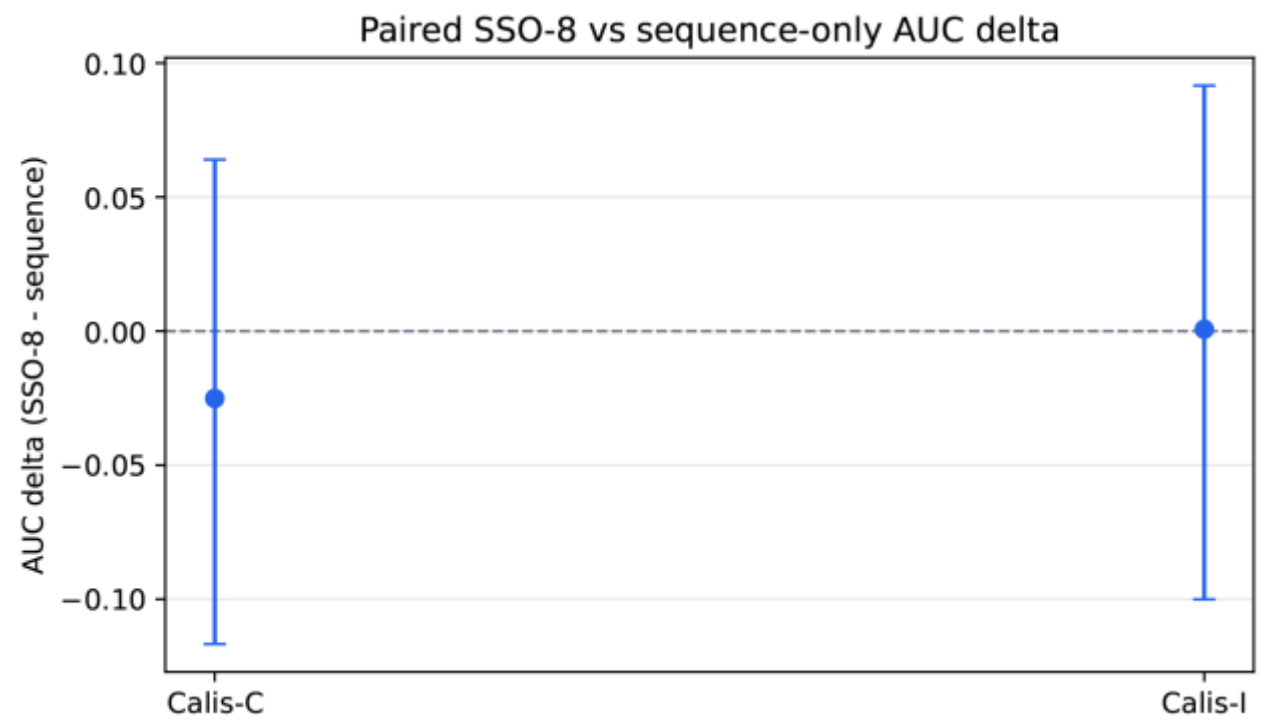

Supplementary Figure S3. Paired SSO-8-minus-sequence AUC deltas with peptide/protein-level bootstrap confidence intervals. The intervals cross zero for both Calis-C and Calis-I.

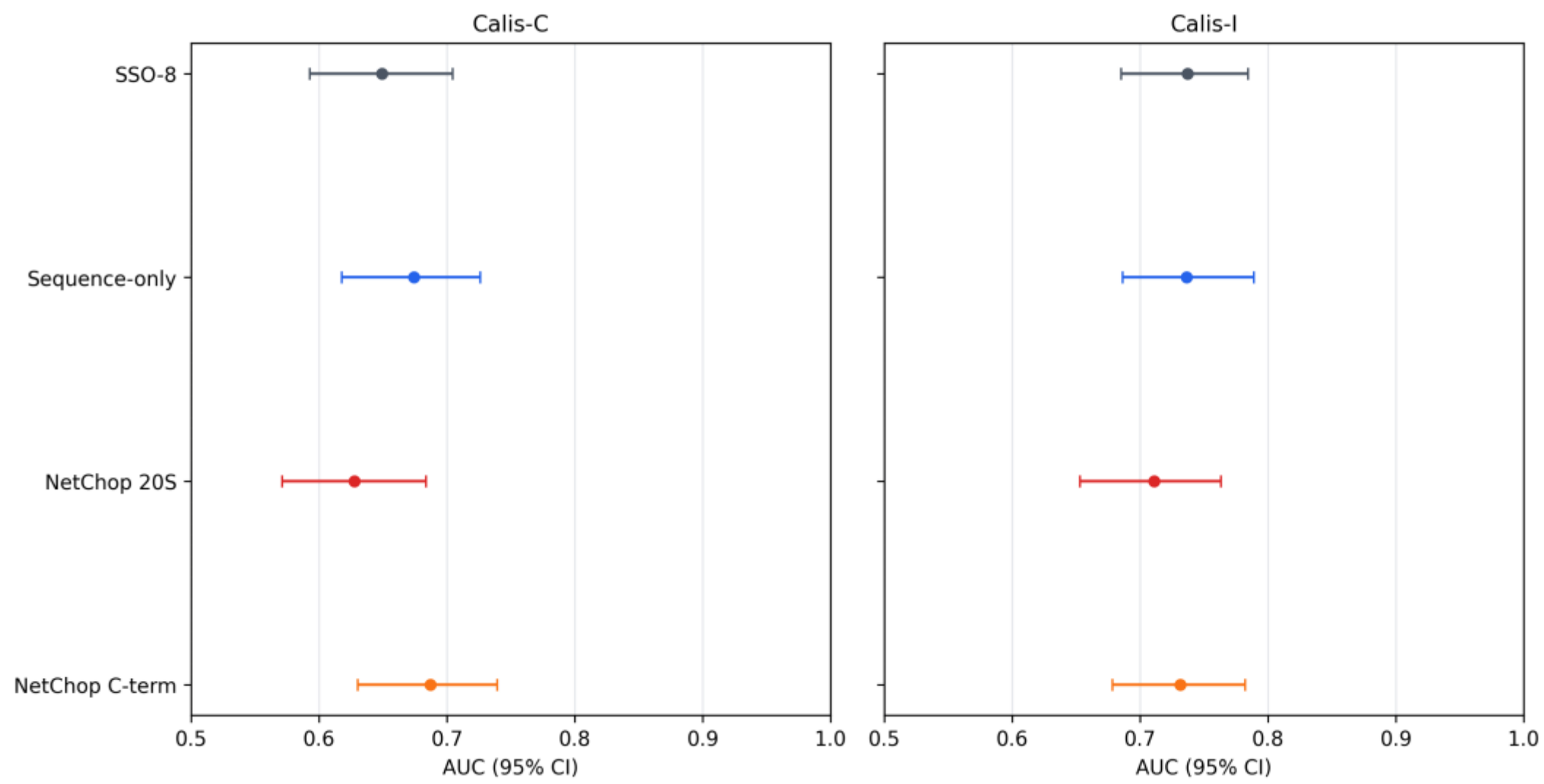

Supplementary Figure S4. All-site Calis AUC comparison for SSO-8, matched sequence-only, and NetChop models. The 20-protein experimental digestion dataset was used for SSO-8 model training, so Calis-C and Calis-I are held-out validation benchmarks.

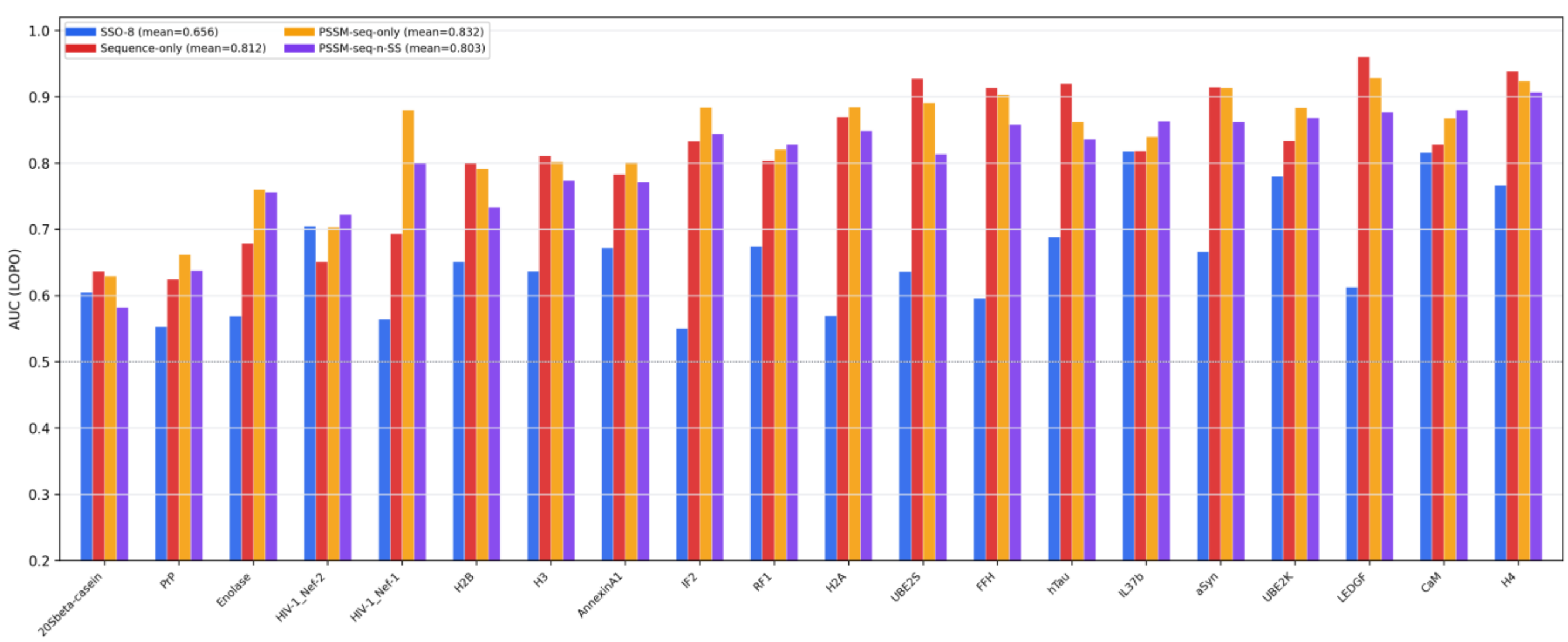

Supplementary Figure S5. Leave-one-protein-out (LOPO) grouped cross-validation on the 20-protein experimental digestion training set. Each fold withholds one parent protein, and bars show per-protein AUCs for SSO-8, sequence-only, and PSSM comparisons. Sorted by mean AUC across methods.

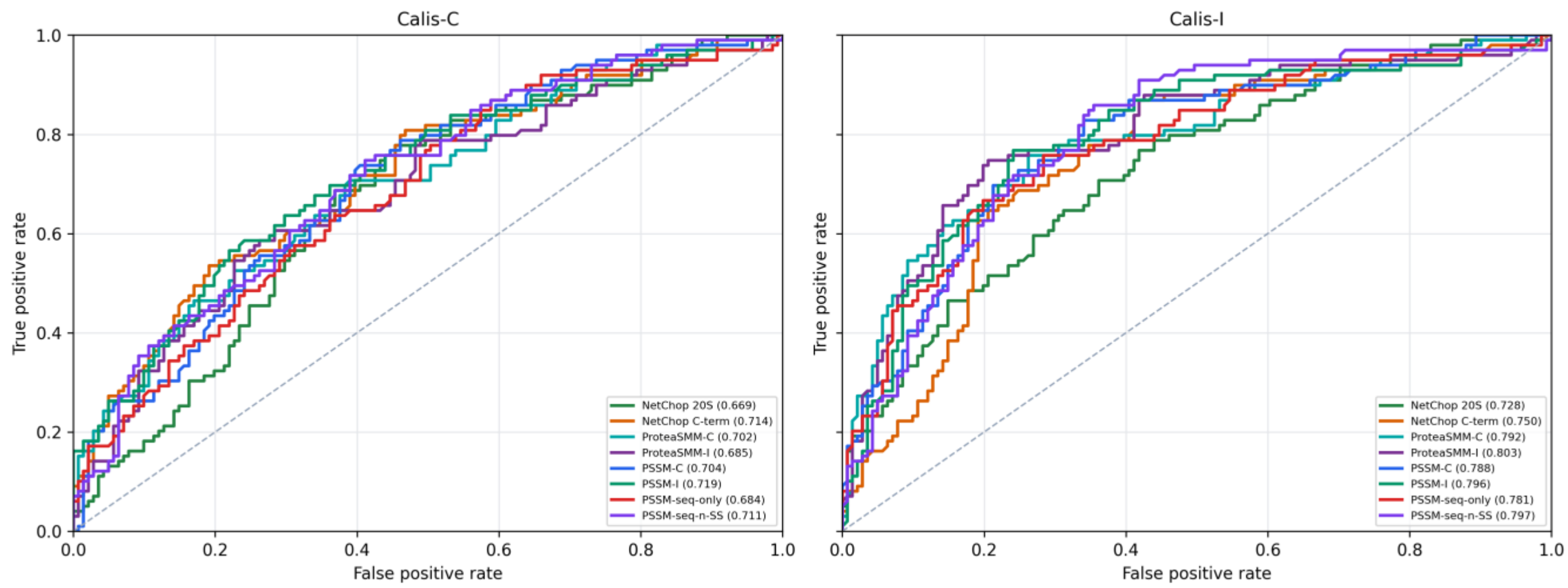

Supplementary Figure S6. ROC comparison on ProteaSMM-comparable Calis residues. Terminal residues that cannot be scored by ProteaSMM are excluded. Same Calis subset used for all methods.

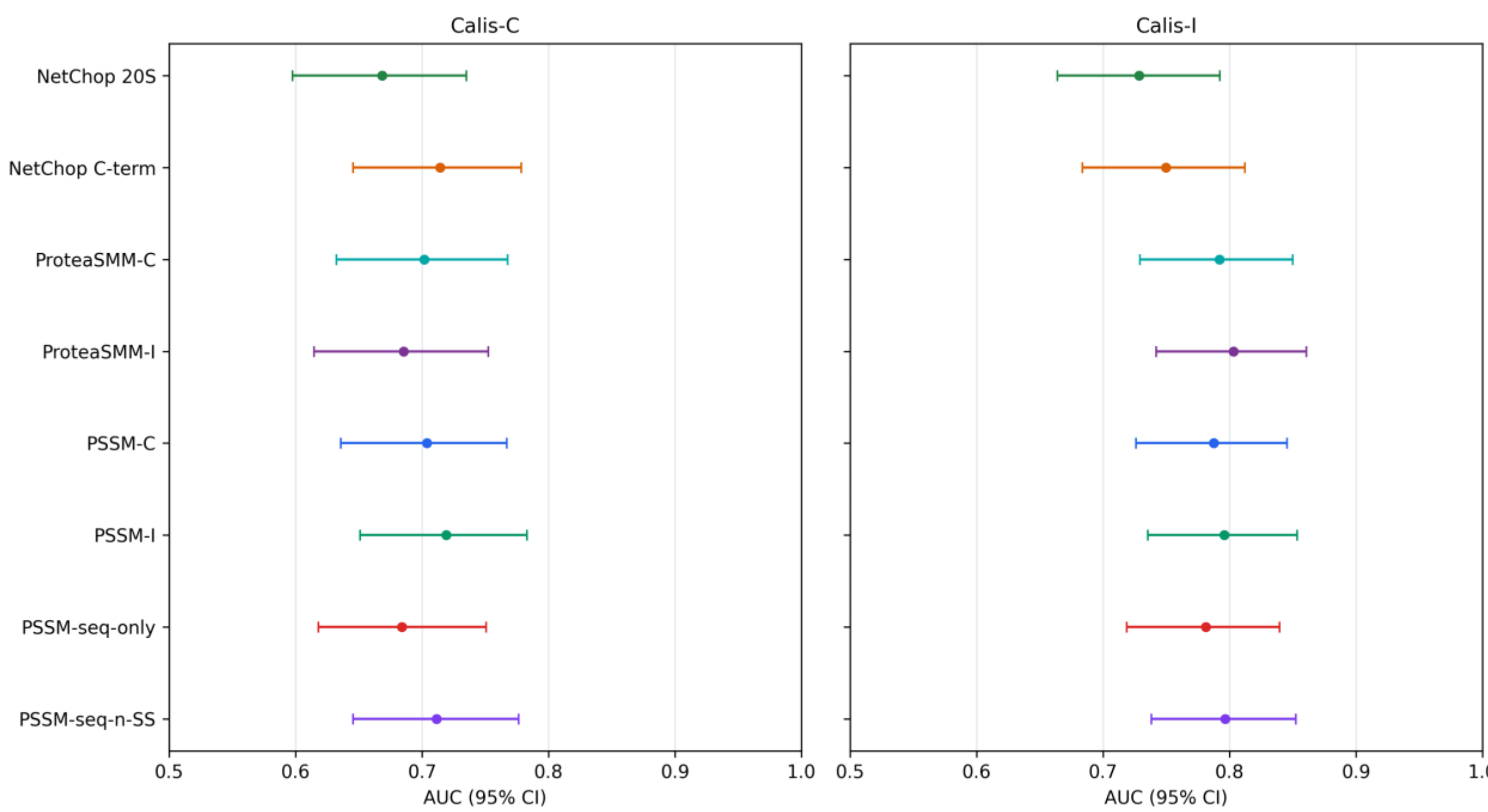

Supplementary Figure S7. AUC point-range comparison on ProteaSMM-comparable Calis residues. The same terminal-excluded Calis subset is used for all methods. AUC values with bootstrap confidence intervals.

Supplementary Dataset 1. Data Sheet with PDB codes for structures included in the PDB corpus.

Supplementary Table S1. Protein structures used for secondary-structure enrichment analysis of proteasomal cleavage sites. Experimentally determined cleavage maps from Weeder et al. (2021) and Soh et al. (2024) were mapped against experimental PDB structures and full-length predicted structures (AlphaFold2 v6 or ESMFold v1). Mapping was performed by pairwise sequence alignment of the structure-derived sequence to the digestion-experiment sequence, followed by STRIDE secondary-structure assignment.

Protein	Source	Length	PDB	Predicted	Sites	Mapped PDB	Mapped predicted
Enolase	Weeder 2021	437	1EBG	AlphaFold2	138	138 (100%)	138 (100%)
PrP	Weeder 2021	211	9DMY	AlphaFold2	59	42 (71.2%)	59 (100%)
HIV-1 Nef fragment	Weeder 2021	30	4EMZ	ESMFold	15	15 (100%)	15 (100%)
HIV-1 Nef full length	Weeder 2021	206	4EMZ	ESMFold	79	64 (81.0%)	79 (100%)
beta-casein	Weeder 2021	224	-	AlphaFold2	38	-	38 (100%)
Annexin A1	Soh 2024	352	4BYN	AlphaFold2	71	47 (66.2%)	71 (100%)
alpha-synuclein	Soh 2024	140	-	AlphaFold2	28	-	28 (100%)
Calmodulin	Soh 2024	148	1CLL	AlphaFold2	30	30 (100%)	30 (100%)
FFH/SRP54	Soh 2024	453	2XXA	AlphaFold2	91	84 (92.3%)	91 (100%)
Histone H2A	Soh 2024	130	1KX5	AlphaFold2	26	25 (96.2%)	26 (100%)
Histone H2B	Soh 2024	123	1KX5	AlphaFold2	25	24 (96.0%)	25 (100%)
Histone H3	Soh 2024	136	1KX5	AlphaFold2	28	28 (100%)	28 (100%)
Histone H4	Soh 2024	103	1KX5	AlphaFold2	21	21 (100%)	21 (100%)
hTau	Soh 2024	441	-	AlphaFold2	89	-	89 (100%)
IF2	Soh 2024	890	3JCJ	AlphaFold2	178	101 (56.7%)	178 (100%)
IL-37b	Soh 2024	173	5HN1	AlphaFold2	35	34 (97.1%)	35 (100%)
LEDGF	Soh 2024	533	6S01	AlphaFold2	107	14 (13.1%)	107 (100%)
RF1	Soh 2024	360	2B3T	AlphaFold2	72	60 (83.3%)	72 (100%)
UBE2K	Soh 2024	200	1YLA	AlphaFold2	40	40 (100%)	40 (100%)
UBE2S	Soh 2024	222	1ZDN	AlphaFold2	45	33 (73.3%)	45 (100%)
				Total	1,215	800 / 1,060 (75.5%)	1,215 / 1,215 (100%)

IDPs (beta-casein, alpha-synuclein, hTau) lack representative soluble experimental structures and were excluded from the PDB-structure panel but included in the predicted-structure panel. LEDGF PDB 6S01 chain K covers only the integrase-binding domain, yielding low resolved-site coverage. Histone structures derive from PDB 1KX5; chains were extracted separately for H2A, H2B, H3, and H4. HIV-1 Nef is absent from the AlphaFold Database, and was instead modeled with ESMFold v1. The PDB-mapped denominator (1,060) excludes the three IDPs with no PDB structure.

Supplementary Table S2. Benchmark comparison summary for SSO-8-MLP-HEC, sequence-only MLP, reference predictors, and PSSM variants on Calis and Soh benchmarks.

Classifier	Calis-I AUC	Calis-C AUC	Soh 2024 AUC
SSO-8-MLP-HEC	0.726	0.650	0.656
Seq-only-MLP	0.678	0.589	0.682
NetChop C-term	0.731	0.687	0.784
NetChop 20S	0.711	0.628	0.534
ProteaSMM-I	0.689	0.644	0.765
ProteaSMM-C	0.684	0.651	0.815
PSSM-seq-only-MLP	0.737	0.634	0.710
PSSM-seq-n-SS-MLP-HEC	0.746	0.651	0.737

Supplementary Table S3. Benchmark comparison summary. AUC values are shown for SSO-8 and NetChop models on all scoreable Calis sites. Sequence-only, matched sequence-only control to SSO-8.

Classifier	Calis-C AUC	Calis-I AUC
SSO-8	0.649	0.737
Sequence-only	0.674	0.736
NetChop 20S	0.628	0.711
NetChop C-term	0.687	0.731

Supplementary Table S4. Predictor performance on ProteaSMM-scoreable Calis residues. Terminal residues that cannot be scored by ProteaSMM are excluded. Sequence-only, matched sequence-only control to SSO-8; PSSM-seq-only, PSSM based on Sequence-only predictions; PSSM-seq-n-SS, PSSM using both Sequence-only and SSO-8; PSSM-C, SSO-8 + NetChop 20S + ProteaSMM-C; PSSM-I, SSO-8 + NetChop C-term + ProteaSMM-I; PSSM-5m, SSO-8 + NetChop 20S + NetChop C-term + ProteaSMM-C + ProteaSMM-I.

Classifier	Calis-C AUC	Calis-I AUC
NetChop 20S	0.669	0.728
NetChop C-term	0.714	0.750
ProteaSMM-C	0.702	0.792
ProteaSMM-I	0.685	0.803
PSSM-seq-only	0.684	0.781
PSSM-seq-n-SS	0.711	0.797
PSSM-C	0.704	0.788
PSSM-I	0.719	0.796
PSSM-5m	0.717	0.806
